# Supplementary material for: Uncovering the structure of self-regulation through data-driven ontology discovery
Source: Nat Commun. 2019 May 24;10:2319. doi: 10.1038/s41467-019-10301-1 (PMC6534563; doi:10.1038/s41467-019-10301-1)
Supplement: Supplementary file 1 — Supplementary Information [file 41467_2019_10301_MOESM1_ESM.pdf]

Supplementary Information

Eisenberg et al.

Supplementary Figures

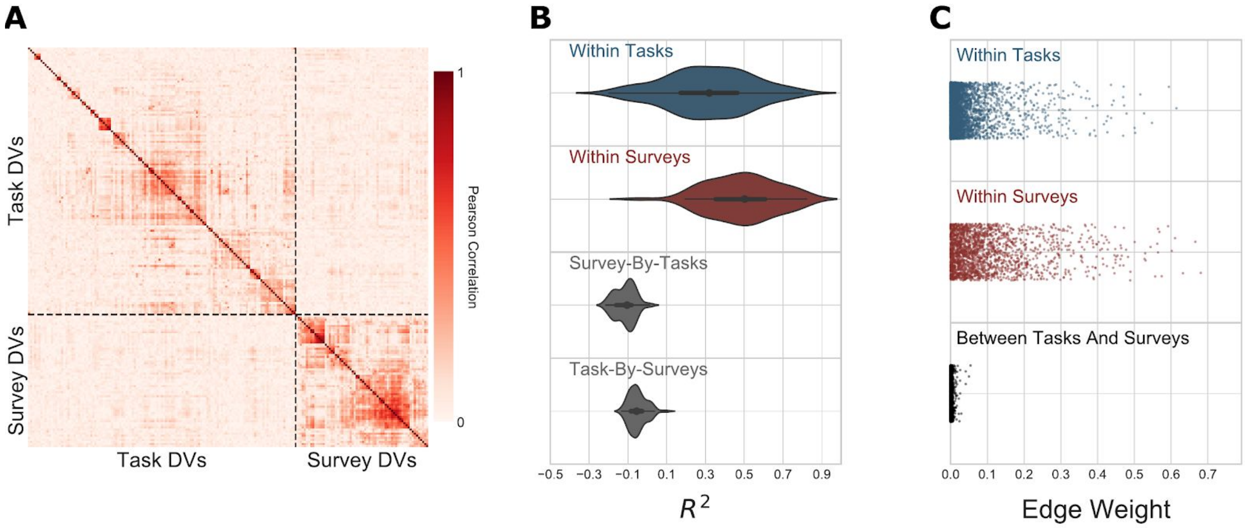

Supplementary Figure 1. Survey-task DV relationships. (a) Pearson correlation between task and survey DVs. DVs are organized by measurement category and ordered based on the respective hierarchical clustering solutions. (b) Cross-validated R^2 derived from cross-validated ridge regression of either a single task or survey DV using all survey or task DVs (holding out the target). (c) Estimate of relationships between or across measurement-category according to the graphical lasso used to estimate Figure 2.

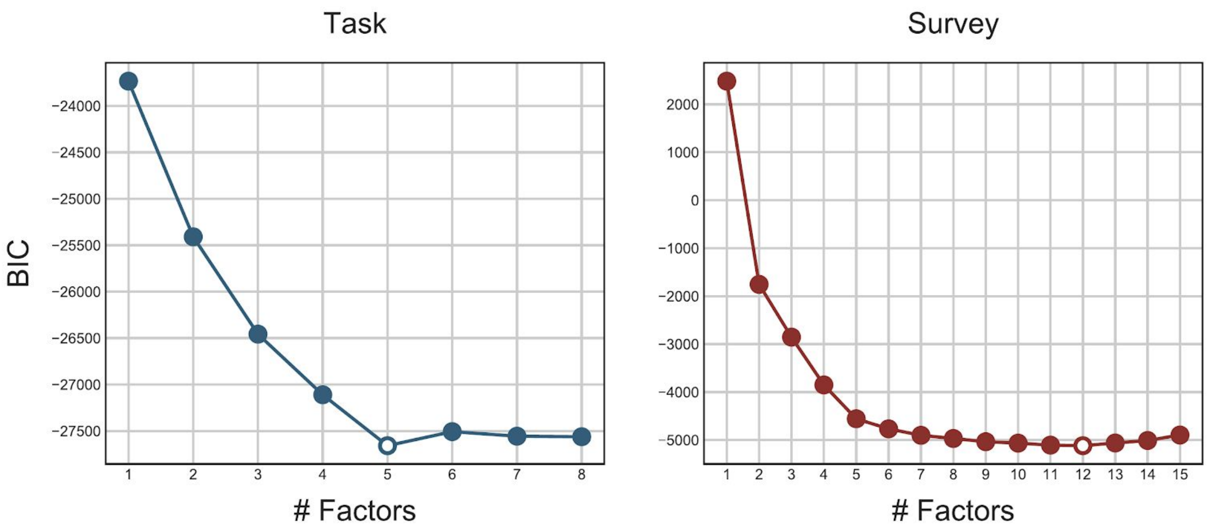

Supplementary Figure 2. Bayesian information criterion (BIC) curves for EFA. BIC was used to determine the optimal number of factors to extract for exploratory factor analysis. The BIC values for a range of factors are shown for surveys and tasks. The optimal dimensionality is indicated by an empty circle.

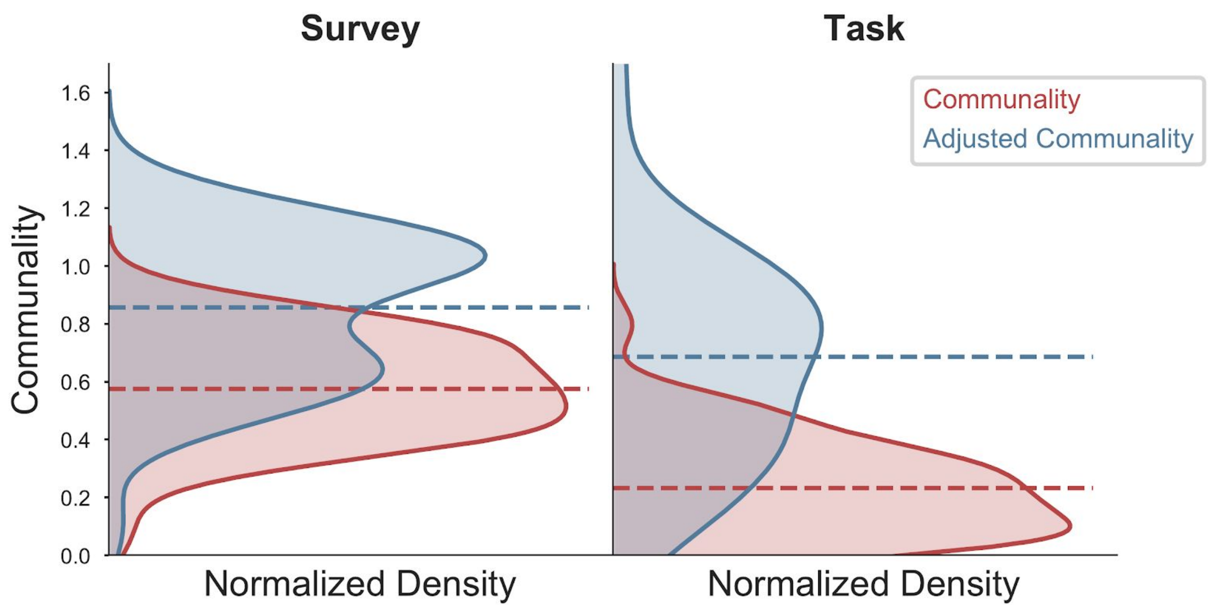

Supplementary Figure 3. Communality correction for test-retest reliability. The distribution of communality (the variance explained by the related EFA model) across DVs is shown in red. The average communality (equivalent to the variance explained of the entire measurement category) is depicted with a red dashed line. Communality was adjusted by dividing by the test-retest reliability, as assessed by Pearson correlation, resulting in the blue distribution. Only DVs with a test-retest reliability above .2 are included in the adjusted distribution. Note that the surveys EFA model performs better than the task EFA model (red curves, survey R^2 = .58, task R^2 = .23), but this difference is attenuated after adjusting for test-retest reliability (blue curves, survey adjusted R^2 = .86, task adjusted R^2 = .68**)**

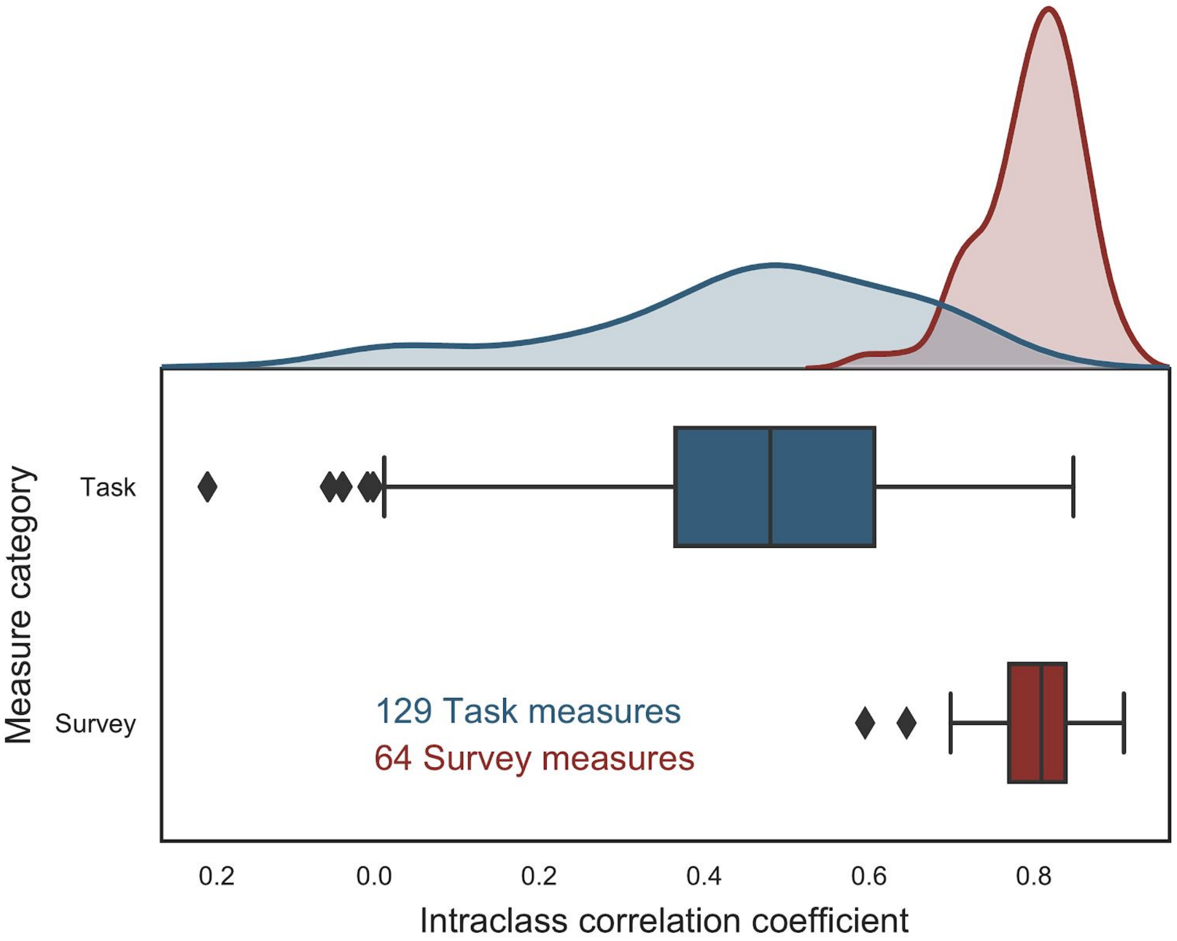

Supplementary Figure 4. Test-retest reliability. Test-retest reliability for the survey and task DVs, as quantified by bootstrapped intraclass correlation coefficient. The full procedure is outlined in Enkavi et al. 2018. Center, median; box limits, upper and lower quartiles; whiskers, 1.5 interquartile range; points, outliers.

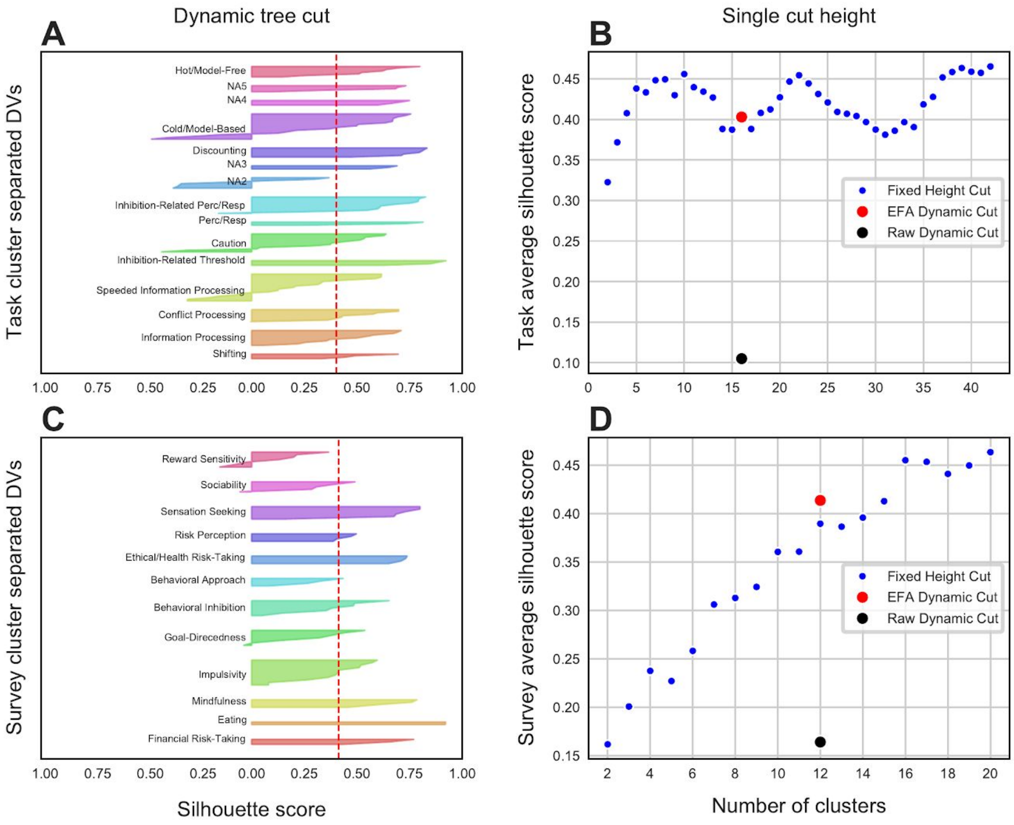

Supplementary Figure 5. Clustering quality assessment using silhouette analysis. (A) and (C) depict the silhouette scores for each DV separated by the clustering solution used in the main paper derived from the DynamicTreeCut algorithm. The average silhouette score for these solutions is depicted using a dashed red line. Note that one task "cluster" consisting of only one DV is not shown. (B) and (D) show the silhouette score using a simpler clustering method - cutting the tree at a single height. The tree was cut at a number of different heights to extract clusters of different sizes (the maximum number of clusters analyzed was a third of the total number of DVs. The silhouette score from the dynamic tree cut solution is shown as a red circle. The dynamic tree cut solution was also used after clustering in "participant space" (see Fig. 1), and the silhouette score for these solutions are shown as black dots.

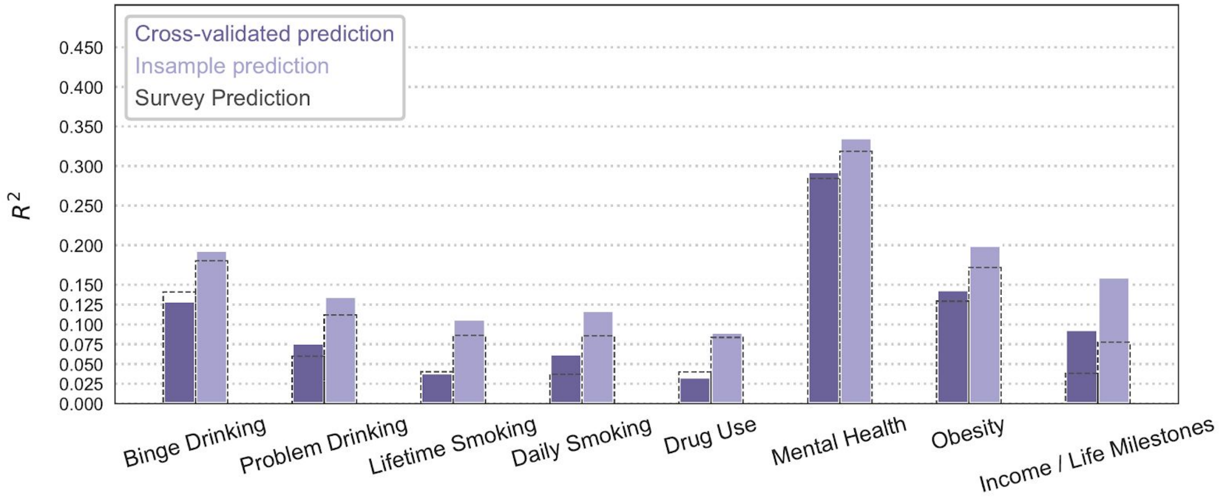

Supplementary Figure 6. Prediction of targets using both task and survey factors. Dashed lines indicate survey prediction performance (identical to Figure 6). All predictions were significant at $p < .05$ based on predictions of shuffled labels.

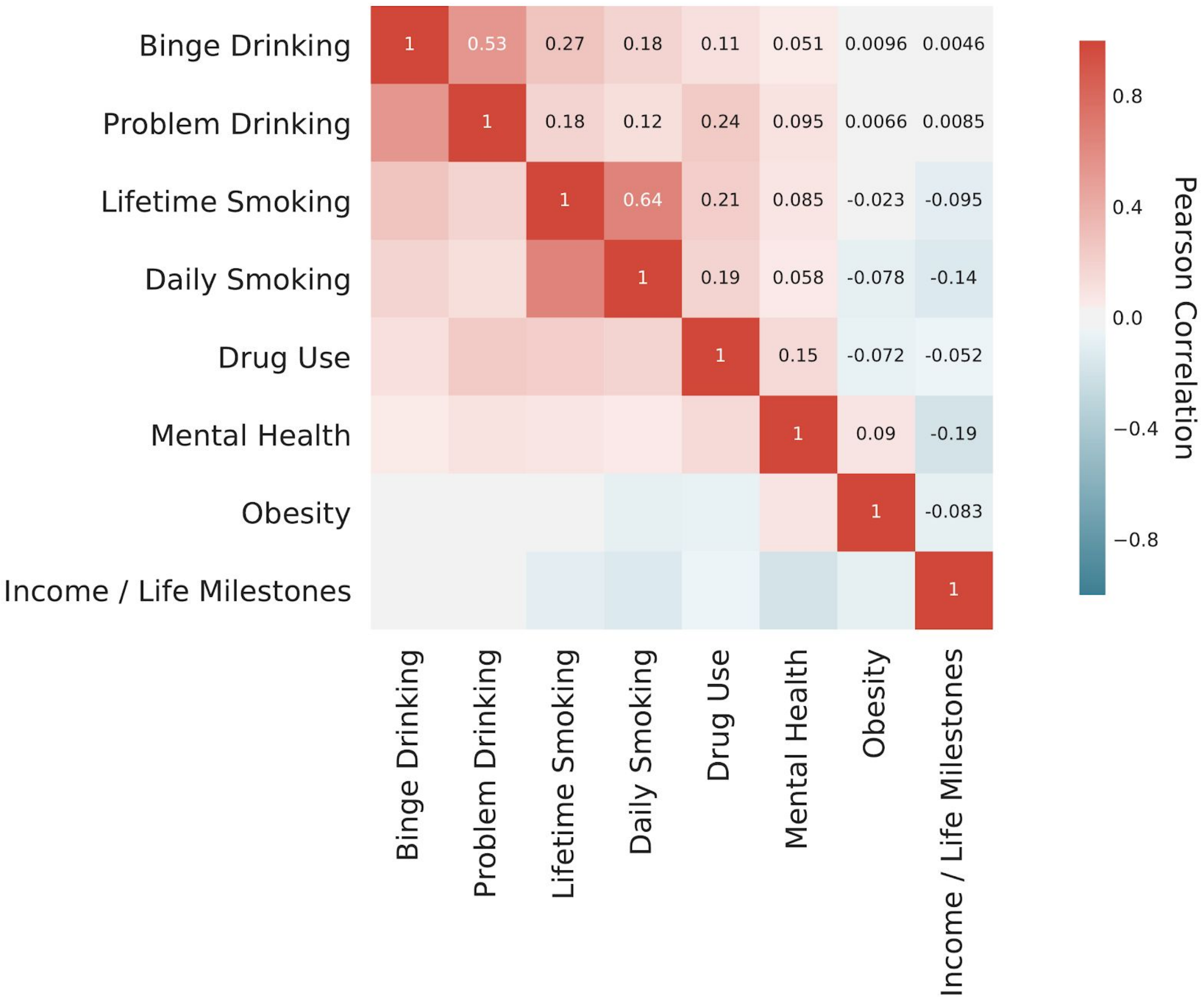

Supplementary Figure 7. Heatmap depicting Pearson correlation amongst outcome target factors.

Supplementary Tables

Supplementary Table 1: List of Self-Report Surveys

**Self-Report Surveys**	**Dependent Variables**	**References**
BIS-11	-Attentional<br>-Motor[1]<br>-Non-Planning	[1]
BIS-BAS	-BAS Drive<br>-BAS Fun-Seeking<br>-BAS Reward-Responsiveness<br>-BIS	[2]
Brief Self-Control Scale	-Self-Control	[3]
Dickman's Impulsivity Inventory	-Functional	[4]
DOSPERT (EB/RP/RT)	-Ethical<br>-Financial<br>-Health/Safety (note: EB[1])<br>-Recreational<br>-Social	[5]

Three-Factor Eating Questionnaire (R18)	-Cognitive Restraint<br>-Emotional Eating<br>-Uncontrolled Eating	6
Emotion Regulation Questionnaire	-Reappraisal<br>-Suppression	7
Five Facet Mindfulness Questionnaire	-Acts with Awareness<br>-Describe<br>-Non-Judgment<br>-Non-Reactive<br>-Observe	8
Future Time Perspective	-Future-Time Perspective	9
Grit Scale	-Grit	10
Impulsive-Venturesome Survey	-Venturesomeness	11
Mindful Attention Awareness Scale	-Mindfulness	12
Multidimensional Personality Questionnaire (Control subscale)	-Control[2]	13
Selection Optimization Compensation	-Elective Selection<br>-Loss-based Selection<br>-Compensation	14

	-Optimization[2]	
Sensation Seeking Survey	-Boredom Susceptibility<br>-Disinhibition<br>-Experience Seeking<br>-Thrill/Adventure Seeking	[15]
Short Self-Regulation Survey	-Control	[16]
Ten Item Personality Questionnaire	-Agreeableness<br>-Conscientiousness[2]<br>-Emotional Stability<br>-Extraversion<br>-Openness	[17]
Theories of Willpower	-Endorse Limited Resource	[18]
Time Perspective Survey	-Past Positive<br>-Past Negative<br>-Present Hedonistic<br>-Present Fatalistic<br>-Future	[19]
UPPS+P	-Lack of Perseverance<br>-Lack of Premeditation	[20]

	-Negative Urgency<br>-Positive Urgency<br>-Sensation Seeking	

[1] Log transformed due to high positive skew (skew > 1)

[2] Reflected and log transformed due to high negative skew (skew < -1)

Supplementary Table 2: List of Behavioral Tasks

**Task**	**Dependent Variables**	**References**
Adaptive N-Back	DDM Parameters[1]<br>Drift Rate as a function of load<br>Average load[3]	[21,22]
Angling Risk Task	Two Conditions (Keep, Release):<br>Adjusted Clicks<br>Coefficient of Variation[3(release condition)]<br>Score[2]	[23,24]
Attention Network Task	DDM Parameters[1, 4 (non-decision)]<br>Alerting Effect<br>Orienting Effect<br>Conflict Effect[4]	[25]
Bickel Titrator	Discount Rate for three payout magnitudes[3]	[26]

Choice Reaction Time	DDM Parameters[1,3(non-decision)]	
Cognitive Reflection Task	Correct Proportion<br>Intuitive Proportion	[27,28]
Columbia Card Task Cold/Hot	Average # of cards chosen<br>Gain Sensitivity[3]<br>Loss Sensitivity[4]<br># Loss Cards Sensitivity<br>Level of Information Use	[29]
Dietary Decision Task	Health Sensitivity<br>Taste Sensitivity	[30]
Digit Span	Forward Span<br>Reverse Span	[31]
Directed Forgetting	-DDM Parameters[1]<br>-Proactive Interference[3]	[32]
Discount Titrator	-Percent Patient	[33]
Dot Pattern Expectancy	-DDM Parameters[1,3 threshold)]<br>-AY-BY<br>-BX-BY<br>-D-prime	[34]

	-Bias	
Go-NoGo	-D-prime<br>-Bias	
Hierarchical Learning Task	-Total Score	[35]
Holt & Laury	-Percent Patient<br>-Beta (inverse softmax temperature)[3]<br>-Risk Aversion (value function curvature)<br>-# Safe Choices	[36]
Information Sampling Task	Two conditions (Decreasing Win, Fixed Win):<br>-Probability Correct at choice<br>-Motivation	[37]
Keep Track Task	-Score	[38,39]
Kirby	-Discount Rate for three payout magnitudes[2(medium magnitude dropped), 3]<br>-Percent Patient Choices[2]<br>-Percent Patient Choices for three payout magnitudes[2]	[40]

Local-Global	-DDM Parameters[1]<br>-Switch Cost<br>-Conflict Effect<br>-Global Bias	38,39
Motor Selective Stop Signal	-DDM Parameters[1, 3 (threshold), 4 (non-decision)]<br>-SSRT<br>-Reactive Control<br>-Selective Proactive Control<br>-Proactive Control	41
Probabilistic Selection Task	-Positive Learning Bias<br>-Value Sensitivity[2]	42
Psychological Refractory Period	-Slope of PRP function	43
Raven's Progressive Matrices	-Score	44
Recent Probes	-DDM Parameters[1]<br>-Proactive Interference	32
Shape Matching Task	-DDM Parameters[1, 3 (threshold), 4(non-decision)]<br>-Stimulus Interference	45
Shift Task	-Accuracy	46,47

	-Learning Rate<br>-Learning to Learn<br>-Model Parameters:<br>- Beta (inverse softmax temperature)[3]<br>- Attentional Decay[4]<br>- RL Learning Rate	
Simon Task	-DDM Parameters[1]<br>-Simon Effect	48
Simple Reaction Time	-Average Reaction Time[3]	
Spatial Span	-Forward Span<br>-Reverse Span	31
Stimulus Selective Stop Signal	-DDM Parameters[1, 3 (threshold), 4 (non-decision)]<br>-SSRT<br>-Reactive Control	49
Stop Signal	-DDM Parameters[1, 3 (threshold), 4(non-decision)]<br>-SSRT (low stop signal probability condition)<br>-SSRT (high stop signal probability condition)[3]<br>-Proactive SSRT speeding<br>-Proactive Slowing	50

Stroop	-DDM Parameters[1,3 (threshold)]<br><br>-Stroop Effect	38,39
Cue/Task-Switch	-DDM Parameters[1, 3 (threshold)]<br><br>-Stimulus Switch Cost<br><br>-Task Switch Cost	51
Tower of London	-Average Move Time[3]<br><br>-# Extra Moves<br><br>-# Optimal Solutions<br><br>-Planning Time	52
Two-step Decision	-Model-Based Index<br><br>-Model-Free Index<br><br>-Perseverance	53
Writing Task	-Sentiment Analysis:<br><br>-Positive Probability<br><br>-Negative Probability	

[1] DDM Parameters include drift rate, threshold and non-decision time

[2] Dropped due to high ($r > 0.85$) correlations with another DV in the same measure

[3] Log transformed due to high positive skew (skew > 1)

[4] Reflected and log transformed due to high negative skew (skew < -1)

Supplementary Table 3: Prediction results using factor scores

	Binge Drinking	Problem Drinking	Drug Use	Lifetime Smoking	Daily Smoking	Mental Health	Obesity	Income/ Life-outcomes
**Task: Ridge[2]**	**R^2 = -0.0 (.01)[1] MAE = .79 (.78)**	**R^2 = -0.1 (.01) MAE = .58 (.57)**	**R^2 = -.02 (.01) MAE = .51 (.51)**	**R^2 = .01 (.02) MAE = .93 (.93)**	**R^2 = .02 (.04) MAE = .82 (.81)**	**R^2 = -0.1 (.01) MAE = .79 (.78)**	**R^2 = .03 (.05) MAE = .87 (.86)**	**R^2 = .06 (.08) MAE = .76 (.76)**
Task: Lasso	R^2 = -.01 (.01) MAE = .79 (.78)	R^2 = -.01 (.00) MAE = .58 (.58)	R^2 = -.00 (.00) MAE = .51 (.50)	R^2 = .01 (.02) MAE = .94 (.93)	R^2 = .02 (.04) MAE = .82 (.81)	R^2 = -.00 (.00) MAE = .79 (.79)	R^2 = .03 (.05) MAE = .87 (.86)	R^2 = .04 (.08) MAE = .77 (.76)
Task: Random Forest	R^2 = -.23 (1.00) MAE = .86 (.00)	R^2 = -.26 (1.00) MAE = .66 (.00)	R^2 = -.25 (1.00) MAE = .63 (.00)	R^2 = -.19 (1.00) MAE = .95 (.00)	R^2 = -.12 (1.00) MAE = .84 (.00)	R^2 = -.26 (1.00) MAE = .87 (.00)	R^2 = -.19 (1.00) MAE = .91 (.00)	R^2 = -.11 (1.00) MAE = .83 (.00)
Task: SVM	R^2 = -.13 (-.12) MAE = .74 (.73)	R^2 = -.10 (-.09) MAE = .44 (.44)	R^2 = -.06 (-.06) MAE = .42 (.41)	R^2 = -.11 (-.09) MAE = .90 (.88)	R^2 = -.26 (-.26) MAE = .71 (.71)	R^2 = -.13 (-.12) MAE = .75 (.74)	R^2 = -.23 (-.23) MAE = .73 (.73)	R^2 = .03 (.05) MAE = .75 (.74)
**Survey: Ridge[2]**	**R^2 = .14 (.18) MAE = .69 (.68)**	**R^2 = .06 (.11) MAE = .57 (.55)**	**R^2 = .04 (.08) MAE = .53 (.51)**	**R^2 = .04 (.09) MAE = .90 (.88)**	**R^2 = .04 (.09) MAE = .80 (.78)**	**R^2 = .28 (.32) MAE = .59 (.58)**	**R^2 = .13 (.17) MAE = .79 (.77)**	**R^2 = .04 (.08) MAE = .76 (.74)**
Survey: Lasso	R^2 = .14 (.18) MAE = .69 (.68)	R^2 = .07 (.11) MAE = .56 (.54)	R^2 = .04 (.08) MAE = .51 (.50)	R^2 = .04 (.07) MAE = .92 (.90)	R^2 = .02 (.08) MAE = .82 (.79)	R^2 = .28 (.32) MAE = .59 (.58)	R^2 = .12 (.17) MAE = .80 (.77)	R^2 = .03 (.07) MAE = .76 (.74)
Survey: Random Forest	R^2 = .03 (1.00) MAE = .75 (.00)	R^2 = -.20 (1.00) MAE = .64 (.00)	R^2 = -.08 (1.00) MAE = .59 (.00)	R^2 = -.04 (1.00) MAE = .89 (.00)	R^2 = -.14 (1.00) MAE = .86 (.00)	R^2 = .17 (1.00) MAE = .64 (.00)	R^2 = .01 (1.00) MAE = .82 (.00)	R^2 = -.10 (1.00) MAE = .82 (.00)
Survey: SVM	R^2 = .11 (.14) MAE = .68 (.66)	R^2 = -.09 (-.08) MAE = .44 (.43)	R^2 = -.06 (-.05) MAE = .41 (.41)	R^2 = -.07 (-.00) MAE = .87 (.83)	R^2 = -.17 (-.16) MAE = .72 (.70)	R^2 = .25 (.29) MAE = .58 (.56)	R^2 = -.09 (-.09) MAE = .72 (.70)	R^2 = .03 (.06) MAE = .75 (.73)
Task and Survey: Ridge	R^2 = .13 (.19) MAE = .70 (.67)	R^2 = .08 (.14) MAE = .57 (.56)	R^2 = .03 (.09) MAE = .53 (.51)	R^2 = .04 (.11) MAE = .89 (.86)	R^2 = .06 (.12) MAE = .79 (.76)	R^2 = .29 (.34) MAE = .59 (.57)	R^2 = .14 (.20) MAE = .78 (.75)	R^2 = .09 (.16) MAE = .74 (.71)

Task and Survey: Lasso	R^2 = .14 (.19) MAE = .70 (.67)	R^2 = .07 (.13) MAE = .56 (.55)	R^2 = .03 (.08) MAE = .51 (.50)	R^2 = .04 (.09) MAE = .91 (.89)	R^2 = .04 (.10) MAE = .81 (.78)	R^2 = .29 (.33) MAE = .59 (.57)	R^2 = .14 (.19) MAE = .79 (.77)	R^2 = .09 (.16) MAE = .74 (.71)
Task and Survey: Random Forest	R^2 = .03 (1.00) MAE = .74 (.00)	R^2 = -.06 (1.00) MAE = .60 (.00)	R^2 = -.10 (1.00) MAE = .58 (.00)	R^2 = -.01 (1.00) MAE = .89 (.00)	R^2 = -.06 (1.00) MAE = .82 (.00)	R^2 = .17 (1.00) MAE = .65 (.00)	R^2 = -.00 (1.00) MAE = .82 (.00)	R^2 = -.06 (1.00) MAE = .79 (.00)
Task and Survey: SVM	R^2 = .10 (.14) MAE = .68 (.65)	R^2 = -.07 (-.07) MAE = .44 (.43)	R^2 = -.05 (-.05) MAE = .40 (.40)	R^2 = -.06 (.02) MAE = .85 (.80)	R^2 = -.13 (-.09) MAE = .72 (.69)	R^2 = .25 (.29) MAE = .58 (.55)	R^2 = -.06 (-.03) MAE = .71 (.69)	R^2 = .08 (.12) MAE = .73 (.69)

[1] Insample score is displayed in parentheses.
[2] Bolded values are used in Figures 5,6 in the main text.

Supplementary Table 4: Prediction results using DVs

	Binge Drinking	Problem Drinking	Drug Use	Lifetime Smoking	Daily Smoking	Mental Health	Obesity	Income/ Life-outcomes
Task: Ridge	R2 = -.25 (.27)[1] MAE = .88 (.67)	R2 = -.37 (.23) MAE = .76 (.56)	R2 = -.28 (.26) MAE = .73 (.54)	R2 = -.31 (.27) MAE = .98 (.73)	R2 = -.19 (.33) MAE = .89 (.67)	R2 = -.25 (.27) MAE = .86 (.66)	R2 = -.25 (.29) MAE = .93 (.70)	R2 = -.12 (.36) MAE = .84 (.63)
Task: Lasso	R2 = -.01 (.02) MAE = .79 (.78)	R2 = -.01 (-.00) MAE = .58 (.58)	R2 = -.00 (.00) MAE = .51 (.50)	R2 = -.00 (.04) MAE = .95 (.93)	R2 = .04 (.09) MAE = .82 (.79)	R2 = -.00 (.00) MAE = .79 (.78)	R2 = .02 (.06) MAE = .88 (.87)	R2 = .11 (.21) MAE = .75 (.70)
Survey: Ridge	R2 = .18 (.36) MAE = .67 (.59)	R2 = -.03 (.22) MAE = .64 (.55)	R2 = -.06 (.20) MAE = .63 (.54)	R2 = -.01 (.23) MAE = .87 (.77)	R2 = .01 (.23) MAE = .80 (.71)	R2 = .21 (.41) MAE = .62 (.53)	R2 = .06 (.29) MAE = .79 (.69)	R2 = .03 (.24) MAE = .77 (.68)
Survey: Lasso	R2 = .26 (.29) MAE = .63 (.62)	R2 = .10 (.13) MAE = .54 (.53)	R2 = .08 (.10) MAE = .51 (.50)	R2 = .06 (.13) MAE = .90 (.86)	R2 = .07 (.15) MAE = .80 (.76)	R2 = .29 (.35) MAE = .59 (.56)	R2 = .15 (.20) MAE = .79 (.77)	R2 = .08 (.18) MAE = .74 (.71)

[1] Insample score is displayed in parentheses.
Random Forests and SVM categorically performed worse and are not shown.

Supplementary Methods

Some of the methods in this project have been previously documented in a protocol paper summarizing our research program focused on behavior change [54]. For convenience, we have reused text from that paper in this supplement.

Mechanical Turk Data Collection Procedure

The dataset used in this analysis was collected as part of a larger project investigating self-regulation and behavioral change, outlined in our previous protocol paper [54]. Our analysis plan was originally divided into a discovery (N=200) and validation (N=300) cohort. Though a majority of the analysis plan was established prior to unblinding the validation dataset (e.g. the selection of the various DVs, specification of quality control measures), some DVs were changed after unblinding, either due to the discovery of coding errors, or the recognition that we had missed canonical analyses for individual measures. Selection of DVs was not informed by the subsequent structure-discovery analyses. Following collection of the discovery and validation cohorts, we retested a subset of participants (n=150). The retest subset was selected randomly

from the discovery and validation cohort and completed the battery a second time. The battery required roughly 10 hours to complete.

We used Amazon's Mechanical Turk (MTurk) to collect the behavioral data, a platform where workers complete Human Intelligence Tasks (HITs). Due to its length, the behavioral battery required multiple sessions to complete. To support this requirement, we developed the Experiment Factory [55], an infrastructure to deploy behavioral measurements on MTurk. The Experiment Factory presented tasks to participants in a random order, and allowed participants to complete the battery at their own pace, finishing as many or as few tasks as they wanted in each sitting. Participants were required to finish the entire battery within one week of accepting the HIT, but no other restriction was placed on their time. Only adults who had completed 2000 previous HITs with a 95% approval, and were between 18-50 and living in the US were invited to participate, though four participants reported that their age was between 50-60. For completion of the battery, participants were paid $60 plus bonuses from performance on specific tasks averaging $10 for their time (minimum: $65, maximum: $75).

As the behavioral battery was long (both in comparison to other psychology studies and MTurk HITs), reducing attrition was a significant consideration. In order to minimize attrition, a number of steps were taken, including providing comprehensive instructions, follow-up emails, and actively fielding questions on various online message boards for MTurk workers. Also, as an incentive to complete, we created a payment schedule that paid a lower rate if the participant failed to complete all 63 measures in the battery. Together, these steps kept attrition manageable: 84**%** of all participants who enrolled ultimately completed the entire battery. We removed any participants who failed to complete the entire battery (102 out of 662), as well as any who failed

to pass quality checks (see "Quality Checks for Cognitive Tasks", below) and continued recruiting until we achieved our sample size goal for each cohort. Due to over-recruiting to ensure we achieved minimum sample sizes, our final samples were 200 (discovery) and 322 (validation). Finally, completed participants were iteratively solicited to take the entire battery a second time, until 150 completed the battery while passing quality checks. Completed participants were randomly ordered before solicitation, and all participants completed the retest battery within 8 months of the initial test (minimum 60 days, maximum 228 days gap between completions).

Quality Checks for Cognitive Tasks

Participants on MTurk are wholly unsupervised, necessitating procedures to ensure data quality. Quality checks were broadly applied to all cognitive tasks to ensure that (1) response times were not unreasonably fast on average, (2) omitted responses were reasonably low, (3) accuracy on cognitive tasks was reasonably high and (4) responses were sufficiently distributed (i.e. the participant didn't only press a single key). The specific criteria we used differed for some tasks, but in general we required that median response times were longer than 200 ms, no more than 25% of responses were omitted, accuracy was higher than 60% and no single response was given more than 95% of the time. These thresholds were set based on evaluation using the discovery cohort only, prior to unblinding the validation cohort. Overall, these steps were taken to ensure that participants in our dataset completed the tasks in earnest. Similar checks could not be performed on the self-report surveys or demographic measurements as we did not collect

response time measures and potentially suspect response patterns (e.g. selecting only one response for every item) may be input honestly.

These criteria were used to evaluate each participant/task pair; failure on any check led to removal of that particular task's data for that participant. In addition, we removed a participant's entire dataset if they failed on four or more individual tasks (38 out of 560 participants were so removed).

These quality checks were intended as thresholds to screen out participants who were intentionally gaming the HIT. We also used task-specific manipulation checks which evaluated particular performance criteria specific to different tasks, necessary for the interpretability of our derived dependent measures. Failing these manipulation checks led to the removal of that participant's data on the failed task, but did not count towards the four failed tasks that would lead to the entire participant being removed from our study. The tasks that used these additional manipulation checks were the stop signal tasks, probabilistic selection task, and two-step decision task.

Selection of Dependent Variables

From the 37 tasks and 22 surveys, we computed 204 dependent variables (DVs). Each survey was analyzed identically - canonical subscale scores were used as DVs. That is, items were appropriately scored (and reversed, if necessary) and summed or averaged in accordance with individual survey scoring procedures.

The tasks were heterogeneous, preventing a completely generic analysis strategy. Nonetheless, many tasks involved speeded decisions between two alternatives, and are well

characterized by reaction time and accuracy. It is well known that reaction time and accuracy are confounded by the speed-accuracy tradeoff [56], which prompted us to use the drift-diffusion model (DDM). The basic DDM transforms accuracy and reaction time into a drift rate, threshold, and non-decision time, roughly corresponding to performance, response caution (a point along the speed-accuracy tradeoff curve) and stimulus-processing/motor-planning, respectively. We fit the DDM parameters using the hierarchical drift-diffusion model (HDDM). HDDM models the DDM parameters hierarchically, such that individual parameters are assumed to be drawn from a group distribution [57]. This procedure improves data efficiency [58], and has been shown to better capture true parameters when dealing with small datasets, or datasets corrupted by trials influenced by processes other than evidence accumulation (e.g., attentional lapses). Though individual parameter estimates are no longer independent (due to the hierarchy), hierarchical models also have been shown to improve point estimates of individual parameters, and are particularly useful when one is interested in correlations between other traits and the individual parameter estimates [59]. The HDDM also allows DDM parameters to be modeled as a function of various conditions. For example, when modeling the stroop task, we modeled drift rate as a function of conflict condition while keeping the other parameters constant.

Tasks that were not speeded choice tasks were heterogeneous and each analyzed according to its own scientific tradition. The full list of measures is available in Table S1 (surveys) and Table S2 (tasks).

Data cleaning and imputation

Because many of our analyses assume normally distributed variables, we transformed skewed variables (absolute skew > 1). We then removed data that were more than 2.5 times the interquartile range above the third quartile or below the first quartile. Any variable that remained excessively skewed after transformation and outlier removal was dropped. 3 variables were dropped due to non-normality. To ensure we did not have redundant variables in the participant-by-measure data matrix, if any two dependent variables derived from the same task or survey measure were correlated $r > 0.85$, one of the variables was arbitrarily removed. 8 variables were dropped using this criteria. This resulted in a final count of 193 variables.

Finally, our data matrix had missing values due to our quality check procedure. Only 3.1% of the overall data matrix was missing, but these missing values were not uniformly distributed amongst the DVs. Instead, 47% of the DVs had no missing values, while a small subsection (the stop signal tasks, probabilistic selection task and two-step decision task) had substantially more missing values (between 10%-30%) due to the additional quality control measures (manipulation checks) taken on those particular tasks. We imputed the data matrix using R's missForest package [60]. See Table S1 and Table S2 for specification of which variables were transformed or dropped due to these procedures.

Visualization of outlier removal, as well as full distributions for all of the DVs used in this paper are available at: https://ianeisenberg.github.io/Self_Regulation_Ontology/cleaning_visualization.html

Description of Self-Report Surveys

The description of the individual measures borrows text from Enkavi et al. [61]. Many of the measures have also been described on the Science of Behavior Change website, and can be demoed there. In addition, the specific items and coding can be found at the expfactory-survey page, and the survey subscale scoring (the particular items used for each subscale) can be found within the expfactory-analysis repo. Data on individual surveys can be found in the Self Regulation Ontology repo.

Behavioral Inhibition and Approach (BIS/BAS)

Developed by Carver and White [2] to measure behavioral approach and inhibition systems, BIS/BAS is a 24 item scale that has a four factor solution: 4 items for BAS drive ("I go out of my way to get things I want."), 4 items for BAS fun seeking ("I'm always willing to try something new if I think it will be fun."), 5 items for BAS reward responsiveness ("When I'm doing well at something I love to keep at it.") and 7 items for BIS ("Even if something bad is about to happen to me, I rarely experience fear or nervousness"). Questions were presented with four point scales.

Barratt Impulsiveness Scale (BIS-11)

BIS-11 [1] is a 30 item questionnaire using a four point scale for short questions. Factor analyses revealed six first order factors that could be further grouped into three second order factors. The first order factors were:

1) attention ("I 'squirm' at plays or lectures")

2) cognitive stability (“I often have extraneous thoughts when thinking”),

3) motor (“I act ‘on impulse’”)

4) perseverance (“I change residences”)

5) the self-control (“I am a careful thinker”)

6) cognitive complexity (“I like to think about complex problems”)

Attention and cognitive stability composed the second order attentional factor, motor and perseverance composed the second order motor factor, while self-control and cognitive complexity composed the non-planning second order factor.

Brief Self-Control scale (BSCS)

BCSC [3] is a 13 item scale presented with 5 response options (1: Not at all to 5: Very much) that measures self-control. An example item is "I am good at resisting temptation."

Dickman's Functional and Dysfunctional Impulsivity

This survey [4] distinguishes between two types of tendencies to act without forethought: one that has negative consequences (dysfunctional) and one that is more optimal (functional). The dysfunctional impulsivity factor consists of 12 true/false items (e.g. "Often, I don't spend enough time thinking over a situation before I act." or "I often say and do things without considering the consequences”) and the functional impulsivity factor consists of 11 true/false items (e.g. "I don't like to do things quickly, even when I am doing something that is not very difficult." or "I don't like to make decisions quickly, even simple decisions, such as choosing what to wear, or what to have for dinner’).

Domain specific risk taking (DOSPERT - RT/RP/EB)

DOSPERT (Domain Specific Risk Taking) survey attempts to capture a more comprehensive, interpretable and translatable construct of risk attitude that is reduced to a single number across domains and confounds marginal value for outcomes and attitudes towards risk in frameworks based on the expected utility theory. The abbreviated version [5] consists of 30 scenarios that are presented with slight variations in question wording to form three separate subscales intended to detangle these. In the risk taking (RT) subscale participants are asked the likelihood they would engage in the described activity; in the risk perception (RP) subscale they are asked how risky they assess each situation to be; and finally in the expected benefits (EB) subscale they are asked the benefit they would expect from each situation. These scenarios were chosen from five domains based on prior literature: Financial (F; "Betting a day's income at the horse races." This consists of two factors: Investing and gambling), health/safety (HS; "Drinking heavily at a social function."), recreational (R; "Going camping in the wilderness."), ethical (E; "Taking some questionable deductions on your income tax return."), social (S; "Admitting that your tastes are different from those of a friend."). All items were presented with a 7 point scale.

Emotion Regulation Questionnaire (ERQ)

Developed by Gross and John [7], the ERQ is a ten item survey that measures two emotion regulation strategies: reappraisal ("I control my emotions by changing the way I think about the situation I'm in") and suppression ("I control my emotions by not expressing them"). Items were presented on a seven point scale.

Five Facet Mindfulness Questionnaire (FFMQ)

FFMQ is a result of a broad psychometric analysis of multiple mindfulness questionnaire. Baer et al. [8] chose the 39 items that best loaded on the five factor solution. The five facets resulting from factor analyses were observing ("When I'm walking, I deliberately notice the sensations of my body moving."), describing ("I'm good at finding the words to describe my feelings."), acting with awareness ("I find it difficult to stay focused on what's happening in the present."), non-judging of inner experience ("I criticize myself for having irrational or inappropriate emotions.") and non-reactivity to inner experience ("I perceive my feelings and emotions without having to react to them."). Items were presented with a five point scale.

Future Time Perspective (FTP)

Developed by Carstensen and Lang [9] in the context of socioemotional selectivity theory and related to the SOC questionnaire, the FTP aims to quantify the age related changes in how people view their future in selecting their goals. It consists of 10 items presented on a five point scale. Based on their scores, people were categorized into having either more open-ended or more limited time perspectives. Older people tend to have the latter. Example items include "Many opportunities await me in the future" and "Most of my life (still) lies ahead of me".

Grit Scale (GRIT-S)

Developed by Duckworth and Quinn [62] the short Grit scale aims to measure perseverance. It consists of eight items presented on a five point scale. Grit-S yields a two factor structure:

consistency of interest (“I often set a goal but later choose to pursue a different one”) and perseverance of effort (“I finish whatever I begin”). We used the total score as a single "grit" DV.

I-7 impulsiveness and venturesomeness questionnaire

Following the I-5 and the I-6, the I-7 is the most recent culmination of Eysenck's work in developing an impulsivity questionnaire[11]. Though the scale was conceived to have three components, we used only the 19 items for the impulsiveness factor (e.g. "Are you an impulsive person”) and 16 items for the venturesomeness factor (e.g. "Would you enjoy the sensation of skiing very fast down a high mountain slope?”), while omitting the empathy factor.

Mindful Attention and Awareness Scale (MAAS)

Developed by Brown and Ryan [12], MAAS is a 15 item questionnaire presented on a six point scale. MAAS focuses on the "individual differences in the frequency of mindful states over time." These items loaded onto a single factor. Sample items include "I could be experiencing some emotion and not be conscious of it until some time later." and "It seems I am ‘running on automatic’ without much awareness of what I’m doing.”

Multidimensional Personality Questionnaire (MPQ) Control Scale

The MPQ [13] is a long and comprehensive questionnaire consisting of multiple subscales. We used the 24-item single factor control subscale and adopted the strategy of Whiteside and

Lynam [63]. Typical true/false items for the MPQ are "I am fast and careless." or "I do things on the spur of the moment."

Selection-Optimization-Compensation (SOC) questionnaire

Baltes et al. [14] developed the SOC as measurement tool of a metatheory of life management strategy within lifespan psychology. It is intended to measure four components. Elective selection ("I concentrate all my energy on a few things" vs "I divide my energy among many things") and loss based selection ("When things don't go as well as before, I choose one or two important goals" vs "When things don't go as well as before, I still try to keep all my goals") together constitute the selection component. The other two components are optimization ("I keep working on what I have planned until I succeed" vs "When I do not succeed right away at what I want to do, I don't try other possibilities for very long") and compensation ("When things don't go as well as they used to, I keep trying other ways until I can achieve the same result I used to" vs "When things don't go as well as they used to, I accept it"). Each item presented two scenarios that participants chose between. There were twelve items for each component.

Short self regulation questionnaire (SSRQ)

The 31 item short self regulation questionnaire was developed by Carey, Neal and Collins [16]. An example item is "I have trouble making plans to help me reach goals" and responses were on a 5 point scale.

Ten-Item Personality Inventory (TIPI)

Developed by Gosling, Rentfrow and Swann [17] TIPI measures the Big Five personality traits of extraversion (E; "Extraverted, enthusiastic"), openness (O; "Open to new experiences, complex"), conscientiousness (C; "Dependable, self-disciplined"), agreeableness (A; "Sympathetic, warm"), emotional stability (ES; "Calm, emotionally stable"). Participants rated themselves on combinations of two adjectives in each question using a seven point scale.

Theories of Willpower Scale

Developed by Job, Dweck and Walton [18] the Theories of Willpower Scale measures people's beliefs about willpower and the role of ego depletion in self-control. It consists of 12 items presented with a six point scale. Higher scores indicate stronger beliefs viewing self-control as a limited resource. Half of the items are about strenuous mental activity ("Strenuous mental activity exhausts your resources, which you need to refuel afterwards (e.g., through taking breaks, doing nothing, watching television, eating snacks).") and the other half about resisting temptations ("Resisting temptations makes you feel more vulnerable to the next temptations that come along.").

3 factor Eating Questionnaire (TFEQ-R18)

TFEQ-R18 is a shortened measure by Karlsson et al. [6] capturing eating behavior in both patient and healthy populations. It measures three aspects of eating behavior: cognitive restraint ("I deliberately take small helpings as a means of controlling my weight."), uncontrolled eating ("When I smell a sizzling steak or juicy piece of meat, I find it very difficult to keep from eating, even if I have just finished a meal.") and emotional eating ("When I feel anxious, I find myself

eating.”). Questions were presented on four point scales though the options for the scale rating differed across questions.

UPPS-P

Whiteside and Lynam [63] initially developed the four factor UPPS after administering a wide variety of impulsivity surveys and combining items from each survey that loaded highest to the four factor solution. This was expanded on by Lynam et al. [20] to measure a fifth construct as well. The five factors that constitute the abbreviated name of the questionnaire are:

1) (negative) urgency - 12 item (“I have trouble controlling my impulses”)
2) (lack of) premeditation - 11 item (“I have a reserved and cautious attitude toward life”)
3) (lack of) perseverance - 10 item (“I generally like to see things through to the end”)
4) sensation seeking - 12 item (“I generally seek new and exciting experiences and sensations”)
5) positive urgency - 14 item (“When I am very happy, I can’t seem to stop myself from doing things that can have bad consequences”).

Zimbardo Time Perspective Inventory (ZTPI)

ZTPI [19] aims to measure how people view time and how this may affect their lives in a broader context. It consists of 56 items and uses a 5 point scale. CFAs showed a five factor solution for the survey: Past-positive (PP; "It gives me pleasure to think about the past”), past-negative (PN; "I think about the bad things that have happened to me in the past”), present-hedonistic (PH; "Taking risks keeps my life from becoming boring”), present-fatalistic

(PF; "My life path is controlled by forces I cannot influence"), and future (F; "It upsets me to be late for appointments").

Zuckerman's Sensation Seeking Scale (SSS-V)

This scale [15] is intended to measure the concept of optimal stimulation level. Participants were presented with two scenarios in each question and asked to indicate which they would prefer. Zuckerman [64] identified four factors that the scale measured: boredom susceptibility (BS; "There are some movies I enjoy seeing a second or even a third time" vs. "I can't stand watching a movie that I've seen before"), disinhibition (D; "I like 'wild' uninhibited parties" vs "I prefer quiet parties with good conversation"), experience seeking (ES; "I dislike all body odors" vs. "I like some for the earthly body smells"), thrill and adventure seeking (TAS; "I often wish I could be a mountain climber" vs "I can't understand people who risk their necks climbing mountains"). We used the 40 item form with ten items for each factor.

Description of Behavioral Tasks

The description of the individual measures borrows text from Enkavi et al. [65]. Many of the measures have also been described on Science of Behavior Change website, and can be demoed there. In addition, the code for individual experiments, which includes information on timing, can be found in the expfactory-experiments repo.

The analysis and post-processing scripts can be found in the expfactory-analysis repo. Data on individual tasks can be found in the Self Regulation Ontology repo.

Adaptive Adjusting Amount Delay Discounting Task

Participants made choices between a fixed large amount at a fixed delay and an immediate amount that started as half the delayed amount and was adjusted either up or down depending on whether the participant chose patiently or impatiently in each trial. The amount of adjustments started at half the immediate amount and was halved at each adjustment. This was repeated for five choices for each fixed later delay and for seven different later delays. The last choice in the procedure was used to estimate the participant's hyperbolic discount rate (or Effective Delay at 50%). One random trial was chosen and contributed to the total bonus the participant received (note the receipt of this bonus was not linked to their chosen delay in any way).

Behavior was evaluated calculating both a hyperbolic discount rate and area under the (discount) curve for each of the three amounts. We determined the decayed value of the fixed larger amount at each delay using the switch point for the set of seven choices for each delay. These decayed values were fit to a hyperbolic function to calculate the discount rate and to calculate the area under the curve connecting them.

Adaptive N-back Task

Participants saw a stream of letters on the screen one at a time. Subjects decided whether the letter on the screen matched the letter N number of trials ago, where N takes on values between 1 and ∞ and was specified at the beginning of each block. Subjects were instructed to press one button when the current letter was the same as the letter that occured N trials ago and another for all other letters. The case of the letters did not matter. Each block consisted of twenty plus the load number of letters. The load was increased if the participant made fewer than three

mistakes in the previous block. It was decreased if the participant made more than five mistakes. Each participant went through twenty blocks.

To evaluate performance across the whole experiment, we calculated the mean load across all blocks. In addition, we used trial-by-trial reaction time and accuracies to calculate individual DDM parameters (drift rate, threshold and non-decision time) using HDDM fit across all participants. In addition, we fit the HDDM with load as a parametric predictor of drift rate.

Angling Risk Task

In this task, which is an extension of the more widely used Balloon Analogue Risk Task [66], participants played a fishing game for thirty rounds in two conditions. In each round their goal was to catch as many red fish as they can, which translated to earnings in that round. There was also one blue fish in each round; if they catch the blue fish, the round ends and they lose all points for the round. They can end the round whenever they want before catching a blue fish to cash out their earnings for the round.

In the original task, there were two weather conditions: a "sunny" condition where participants could always see how many fish there were in the lake, and a "cloudy" condition where they could not. Due to time constraints on the total length of our task battery we only used the "sunny" condition.

There were also two release rules. In the "keep" condition each red fish the participants caught stayed out of the lake (sampling without replacement and increasing the probability of catching a blue fish after each draw). In the "release" condition the red fish were thrown back in the lake so the number of fish in the lake remained constant for the whole round. The number of

fish varied between 1 and 200 for each round. Total score on this task contributed to the final bonus each participant received.

We calculated three DVs for each release condition: the adjusted number of clicks (number of clicks on rounds when the blue fish was not caught), the "coefficient of variation" (defined as the standard deviation of the number of clicks on each round when the blue fish was not caught) and the total score in the game. Adjusted clicks and total score were highly correlated, so total score was dropped (see "Data Cleaning and Imputation").

Attentional Network Task

Participants indicated the direction of a center arrow that was surrounded by two flankers on each side. The set of five stimuli (target + flankers) appeared below or above a center fixation cross. There were three conditions depending on the direction of the surrounding arrows: incongruent if flankers were arrows pointed in the opposite direction than the target stimulus; congruent if they were arrows pointed in the same direction and neutral if the flankers were horizontal lines instead of arrows. There were four conditions depending on the cue before the presentation of the target stimuli: In "no cue" trials, no cue was presented before the target stimulus. In "double cue" trials, two simultaneous cues were flashed above and below the fixation cross. In "center cue" trials, the cue was flashed in the location of the fixation cross. In "spatial cue" trials, the cue was flashed in the location where the target stimulus will follow. The cue was a quick flash of a star. Participants completed 24 practice trials and 144 experimental trials (2 (locations) x 4 (cues) x 2 (direction) x 3 (flanker) x 3 (blocks)).

Using trial-by-trial reaction time and accuracies, we calculated individual DDM parameters (drift rate, threshold and non-decision time) using HDDM fit across all participants. In addition, we fit the HDDM with cue-type (informative spatial, double, center, and no cue) and flanker-type (congruent, incongruent) as categorical predictors of drift rate. Differences in drift rate coefficients across conditions provides putative measures of three pillars of attention: alerting (no cue - double cue), orienting (central cue - spatial cue) and executive control (incongruent - congruent flanker). In each case, drift rate was expected to be smaller in the former condition, and greater in the latter condition (e.g. "incongruent - congruent drift rate" is generally negative), analogous to a longer reaction time in the former condition.

Choice Reaction Time

In this task participants saw either orange or blue squares on the screen for each trial. They were instructed to respond using a different button for each stimulus as quickly and accurately as possible. They completed twenty practice trials and three blocks of fifty test trials.

Using trial-by-trial reaction time and accuracies, we calculated individual DDM parameters (drift, threshold and non-decision time) using HDDM fit across all participants.

Cognitive Reflection Task

In the classical version of the task, participants answered three questions that had numeric answers. The questions were worded such that there was a spontaneous, intuitive, but erroneous answer and a correct answer that typically requires a slower and more thoughtful response. Because our sample was likely familiar with the questions of the classical version [67] we used

three items from Toplak, West and Stanovich's [27] as well as three from Primi et al.'s [28] expansions. Two DVs were calculated from this task: the proportion of correct choices, and the proportion of "intuitive" (but incorrect) choices.

Columbia Card Sorting Task

In this task participants played a card game in multiple rounds. Their goal in each round was to collect as many points as possible by flipping cards from a deck of 32. Each deck contained gain and loss cards. The participants gained points for each gain card they chose, and lost points and immediately ended the round if a loss card was chosen. Each gain card was worth either 10 or 30 points while each loss card cost either 250 or 750. There were 1 or 3 three loss cards in each round. All the round information was always on display throughout the round. Participants played 24 rounds in two conditions. In the hot condition they flipped each card individually and saw the outcome of the card immediately whereas in the cold condition they indicated how many card they would want to flip given the round information. After 24 rounds, three random trials were chosen to contribute to the overall bonus the participant received.

The number of cards chosen on each round was modeled as a function of the amount each gain card was worth, the amount lost if the loss card was chosen, and how many loss cards existed. The standardized beta coefficients for these three variables were taken as sensitivity to gain, loss, and probability, respectively. A summary metric of "information use" was also calculated ranging from 0-3, which was the total number of significant ($p < .05$) sensitivity beta coefficients. Finally we also included the average number of cards chosen across all rounds.

Delay Discounting Titrator

In this task, participants chose between a sooner-smaller monetary amount and a larger-later one. Unlike the other two intertemporal choice tasks in our battery, the options in this task were more variable across participants. The sooner reward can be immediate or delayed two weeks. The later reward can be either two or four weeks later than the sooner reward. The sooner amounts were drawn from a normal distribution with a mean of 20 and standard deviation of 10, clipped at 5 and 40. The relative difference between the sooner and later reward can be 1, 5, 10, 15, 20, 25, 30, 50, 75% higher. Participants made 36 choices. One random trial was chosen and contributed to the total bonus the participant received (note: the receipt of this bonus was not linked to their chosen delay in any way).

Behavior from this task was evaluated by both tallying the number of patient choices across all trials and fitting a hyperbolic model to the choices where the subjective value of the delayed amount decreases according to the following function: amount/1+discount rate*delay.

Dietary Decision-making Task

This task consisted of two phases. In the first phase participants rated the healthiness and tastiness of fifty food items on a five point scale. A reference item that fell towards the middle of these ratings was chosen. Specifically, we chose the item that was closest to the median healthiness and tastiness value of all food items. In the second phase they were given a choice between this reference item and the remaining forty nine items and rated whether they would prefer the current item over the reference item on a five point scale (Strong No, No, Neutral, Yes, Strong Yes).

This preference response was modeled as a function of the current item's health and taste ratings. The standardized coefficients for health and taste were taken as measures of "health sensitivity" and "taste sensitivity" and were the two DVs used for this task.

Digit Span

On each trial, participants viewed a series of digits and were instructed to use the mouse to enter the digits either in the order of presentation or in reverse order, on a number pad after the digits disappeared. Participants were asked to report the digits in the order of presentation for the first fourteen trials, and in reverse order for the next fourteen trials. The number of digits started at 3 and increased by 1 if the participant entered the correct series. The number of digits decreased by 1 after two incorrect responses. The forward and reverse span were used as the two DVs for this task.

Directed Forgetting Task

On each trial participants were presented with six letters, three on top of the screen and three on the bottom of the screen, and were instructed to remember all six letters. After the letters disappeared, a cue indicated whether the top or the bottom letters should be forgotten; these letters were known as the forget set. The other three letters were the memory set. After the cue, subjects were presented with a probe - a single letter. Participants indicated whether the probe was in the memory set by pressing one button and another button if not. Trials were either "positive" (the letter was in the memory set), "negative" (the letter was in the "to be forgotten"

set) or "control" (the letter was not shown at all on that trial). Participants completed three rounds of twenty four trials.

Using trial-by-trial reaction time and accuracies, we calculated individual DDM parameters (drift rate, threshold and non-decision time) using HDDM fit across all participants. In addition, we fit the HDDM with probe type (positive, negative, control) as a categorical predictor of drift rate. The difference in drift rate coefficients between negative and control probes (negative - control) is putatively related to proactive interference. Drift rate was expected to be smaller in the former condition, and greater in the latter condition, analogous to a longer reaction time in the former condition.

Dot Pattern Expectancy Task

An adaptation of the AX continuous performance task [68,69], in this task [70], participants saw cue-probe pairs (configurations of dots) on each trial. Each trial consisted of the presentation of one out of six cue stimuli followed by the delayed presentation of one out of six probe stimuli, followed by a response. One pair, consisting of a target cue (A) and a target probe (X), was considered the "target pair" (AX trial) and was identified to the participant at the beginning of the task. Subjects were instructed to press one button if they saw the target pair and another button for all other cue-probe pairs (referred to as "BX", "BY", or "AY"). There were 32 trials in each block and four blocks following a practice block. 68.75% of trials were AX (target) trials, 12.5% were BX, 12.5% were AY, and 6.25% were BY.

Using trial-by-trial reaction time and accuracies, we calculated individual DDM parameters (drift rate, threshold and non-decision time) using HDDM fit across all participants.

In addition, we fit the HDDM with trial type (AX, AY, BX, BY) as a categorical predictor of drift rate. Differences in drift rate between AY and BY trials is putatively related to proactive control (AY - BY), while differences between BX and BY is putatively related to reactive control (BX - BY). We also calculated d' and bias across all trials, which are functions of participant hit rates and false-alarm rates.

Go/no-go Task

In this task participants saw one of two colored squares. They were instructed to respond as quickly as possible by pressing a button for one color and to withhold their response for the other color. Participants completed ten practice trials with feedback and 350 test trials without feedback. 90% of the trials were go stimuli. d` and bias were calculated as the two DVs for this task.

Hierarchical rule learning Task

Participants responded to eighteen different stimuli (varying on 3 shapes, 3 orientations and 2 colors) using one of three buttons. Originally [35], there were two rule sets. In the flat rule set, each stimulus response pairing had to be learned individually. In the hierarchical rule set, a hierarchical relationship between the stimuli and the correct responses allowed a two-step policy where, for example, the color indicated whether the response should depend on the shape or the orientation to be a more efficient strategy. We only included the hierarchical rule set in our implementation. There were 360 trials per rule set. Total score was the only DV calculated and contributed to the bonus the participants received at the end of the experiment.

Holt and Laury Titrator

Participants chose between two gambles for ten questions. One of the gambles was the safe gamble where the two outcomes had low variance ($80 and $100) and the other gamble was the risky gamble where the two outcomes had high variance ($190 and $5). Across the ten questions, the probability of each outcome changed for both gambles. This systematic changing (i.e. titration) of the probabilities was intended to sway participants' choice from the safe to the risky gamble.

We calculated four dependent variables from this task. First we tallied the number of safe choices across the ten gambles. Then we fit the Cumulative Prospect Theory (CPT) as outlined in Toubia et al. [71] to extract three parameters: a risk aversion parameter indicating the curvature of the value function, a probability weighting parameter indicating the curvature of the probability weighting function, and an inverse temperature parameter indicating how much the behavior used CPT versus random choice.

Information Sampling Task

Participants were presented with a five by five grid of gray boxes where each box covered one of two colors. Participants were instructed to indicate which color they thought was the majority (one color made up between 13 and 18 of the boxes). To make this decision, participants were told they could reveal the color of any box by clicking on them. There were two conditions. In the fixed win condition, participants won or lost 100 points depending on the accuracy of their color choice regardless of how many boxes they opened. In the decreasing win

condition, each round began with 250 points and each opened box cost 10 points on the potential winnings of the round. An incorrect choice in this condition also lead to a loss of 100 points. Participants completed ten rounds of each condition. The DVs from this task were the average response latency of opening a box (motivation) and the average probability of making the correct decision in each round (see [37] for derivation) for each condition.

Keep Track Task

Participants were presented with a stream of fifteen words in each round where each word exclusively belonged to one of six categories. Participants were instructed to remember the last word presented in a subset of those categories, which they entered in a textbox at the end of the round. The rounds differed in their difficulty based on the number of categories (ranging from 3-5). Before the task began, participants were given all target categories and all possible words that might appear for each category to avoid any confusion. Each round began by specifying which categories were relevant that round and participants completed three rounds for each of the three difficulty levels. The score for each round was the sum of target words correctly entered into the textbox at the end. The maximum total score was therefore 36 (three repetitions of 3 points for each "3 category" round, 4 points for each "4 category" round and 5 points for each "5 category" round). The total score was the only DV for this task.

Kirby Delay Discounting Items

This is one of the most commonly used intertemporal choice tasks that is based on the multiple price list methodology in the economics literature. Similar to other intertemporal choice

tasks in the battery, participants made choices between smaller immediate monetary amounts and larger delayed monetary amounts. The stimuli were divided into three groups (small, medium, large), depending on the size of larger reward with nine choices in each group. Each of these nine choices spanned the same range of implied hyperbolic discount rates if they were to be the indifference points for a given participant (00.016-0.025) that were spaced equidistantly on a log-scale of hyperbolic discount rates. One random trial was chosen and contributed to the total bonus the participant received (note the receipt of this bonus was not linked to their chosen delay in any way).

The performance from this task was evaluated using two metrics. First we tallied the number of patient choices both for all of the trials as well as for each amount group. Then we calculated the hyperbolic discount rate implied by the switch points for each of the three amount group as well.

Local-global Task

Participants were shown a large letter (either "H", "S", or "O") composed of smaller letters (also either "H", "S", or "O"). In each round, the color of the stimulus directed the participant to attend to either the "global" (large) letter or the "local" (small) letter. They then pressed one of two buttons to indicate whether the attended letter was an "H" or an "S" (the "O" was therefore never a response, and served as a neutral distractor when present).

In the congruent condition the small and large letters matched. In the incongruent condition, the larger letter was composed of smaller letters that would trigger the opposing

response. In the neutral condition the irrelevant letter was "O", which did not trigger an alternative response. Participants completed 96 trials.

Using trial-by-trial reaction time and accuracies, we calculated individual DDM parameters (drift rate, threshold and non-decision time) using HDDM fit across all participants. In addition, we fit the HDDM with condition (global vs local), conflict condition (congruent, incongruent, neutral), and switch condition (whether global/local condition was the same or different as the last trial) as categorical predictors of drift rate. Differences in drift rate between global and local conditions putatively relates to a "global bias" (global - local), differences between conflict conditions reflect a general conflict effect (conflict - non-conflict), and differences between stay and switch trials reflect a task-set switch cost (switch - stay). In each case, drift rate was expected to be smaller in the former condition, and greater in the latter condition (e.g. "switch - stay drift rate" is generally negative), analogous to a longer reaction time in the former condition.

Motor Selective Stop Signal Task

On each trial, participants were shown one out of four stimulus, where each stimuli was associated with one of two responses, either the left or right hand. Participants were instructed to respond to the stimuli as quickly as possible without sacrificing accuracy. After initial practice to familiarize participants with the stimulus-response mapping, subjects were instructed that a red star (stop signal) would appear on some trials. Additionally, participants were informed that one of their responses (left or right hand) was the critical response, which was randomized across

participants. Participants were instructed to stop their response if they saw a red star and were about to respond with their critical hand.

The red star appeared around the stimulus with a delay (stop-signal delay) ranging from 0ms - 850ms after stimulus onset. Stop-signal delay was adjusted during ‘stop’ trials using a one-up, one-down staircase procedure in 50ms increments. Participants completed 5 blocks of 60 trials each. 60% of the trials were "go" trials, 20% were "stop" trials (where the stop signal was shown for the critical hand), and 20% were "ignore" trials (where the stop signal was shown for the non-critical hand).

Stop signal reaction time was calculated based on the "critical" trials, a measure that putatively reflects inhibitory control. Using trial-by-trial reaction time and accuracies on the "go" trials, we also calculated individual DDM parameters (drift rate, threshold and non-decision time) using HDDM fit across all participants. In addition, we fit the HDDM with "critical condition" (critical vs non-critical hand) and non-stop conditions ("go" vs "ignore") as categorical predictors of drift rate. Proactive control was defined as the difference in drift rate between the two critical conditions (critical - non-critical). Reactive control was defined as the difference in drift rate between the non-stop conditions (ignore - go). In each case, drift rate was expected to be smaller in the former condition, and greater in the latter condition, analogous to a longer reaction time in the former condition.

Probabilistic Selection Task

This task was divided into two stages. In the first, participants learned to choose between three pairs of abstract shapes based on their reward probabilities. The probabilities for the shapes

in each pair were 80%/20%, 70%/30% and 60%/40%. Each learning block was 60 trials. Training continued for at least 3 blocks and ended when participants reached a performance criterion (greater than 70% correct on the easiest pair, 65% on the middle pair, and 50% correct on the hardest pair) or 8 blocks had passed, whichever happened first. Following this learning phase, a test phase occured where participants were shown 6 repetitions of novel pairs of stimuli that were not shown during the learning phase (e.g. 80%/30%).

Two DVs were calculated: a general value sensitivity, and a positive learning bias. These were computed based on a logistic regression model that modeled choice (the probability of choosing the left stimulus) during the test phase using the following formula:

$$(1)\ P(left\ choice) = value\ difference * value\ sum - value\ sum + choice\ lag$$

Each stimulus value was computed based on the participant's experience with that stimulus during the training phase (rather than the objective probabilities). "Value sensitivity" was defined as the main effect of value difference. "Positive learning bias" was defined as the interaction between value difference and value sum. That is, some people may be more sensitive to value differences if both stimuli were high value, indicating that they learned the value of the "good" stimuli more effectively than the "bad" stimuli during the learning phase. The alternative could also be possible - participants who learn better from negative feedback (and thus better learn the value of the low-value stimuli) would be more sensitive to value differences when the value sum was low. "Choice lag" was a nuisance variable that captures the tendency for participants to repeat their last response.

Psychological Refractory Period Task

Participants responded to two sequential cues (a colored box was displayed, followed by a number). First they responded using one of two buttons depending on the color of a box. Then they responded using one of two other buttons depending on the number that appeared in the box. The interstimulus interval (ISI) between the two cues could be 50, 150, 300 or 800 ms. Participants completed 32 trials of practice with feedback and 200 test trials without feedback.

The principal effect of interest in the PRP task relates to the idea that processing of the first task slows processing of the second task (either because of a computational "bottleneck" or shared, limited resources; [43]). The PRP effect is the observation that the relationship between reaction time on the second task and the ISI approaches a slope of -1 at short ISI's, implying that no additional benefit was gained from additional exposure time to the cue. We calculated the slope between the second task's reaction time and ISI and used that as our only DV. An unsigned (absolute) slope of less than one could be interpreted as reflecting less resource constraint (i.e., enhanced parallel processing).

Raven's Progressive Matrices

Raven's Progressive Matrices [44] is a common measure of intelligence, specifically fluid intelligence, that is thought to reflect the ability to infer abstract rules and reason about them to solve problems. On each trial participants were asked to choose the item that would complete a pattern. There were 18 items which increased in difficulty. Total number correct was the only DV.

Recent Probes Task

Participants were presented with six letters displayed in two rows. These six letters were called the "memory set". Following the presentation of this memory set, participants were presented with a single letter and asked to indicate whether the single letter was in the memory set using one of two buttons. Half of each memory set was from the previous memory set while the other half was novel. The probes were of four types: member of current memory set but not of last two memory sets (positive-not-recent), member of current memory set and of previous memory set (positive-recent), member of previous memory set but not of current memory set (negative-recent) and member of neither of the last two memory sets (negative-not-recent). Participants complete twenty four trials per run for three runs.

Using trial-by-trial reaction time and accuracies, we calculated individual DDM parameters (drift rate, threshold and non-decision time) using HDDM fit across all participants. In addition, we fit the HDDM with probe type (positive-recent, positive-not-recent, negative-recent, negative-not-recent) as a categorical predictor of drift rate. Differences in drift rate coefficients between the negative conditioned (negative-recent - negative-not-recent) was taken as a measure of proactive interference. Drift rate was expected to be smaller in the former condition, and greater in the latter condition, analogous to a longer reaction time in the former condition.

Shape Matching Task

Participants indicated whether a white shape on the right of the screen and the green shape on the left of the screen were the same using one of two buttons. On half of the trials, a red shape 'distractor' overlaid with the green shape. The correct response did not depend on this red shape. The red shape was identical to or different from the green shape. Participants completed forty trials for seven types of trials depending on the relationship between the target and the probe, target and the distractor, and distractor and the probe.

Using trial-by-trial reaction time and accuracies, we calculated individual DDM parameters (drift rate, threshold and non-decision time) using HDDM fit across all participants. In addition, we fit the HDDM with condition (the seven relationships between the target, probe, and distractor) as a categorical predictor of drift rate. Stimulus interference was calculated as the difference in drift rate when there was a distractor present (that did not match the target or probe) and when there was no distractor present. Drift rate was expected to be smaller in the former condition, and greater in the latter condition, analogous to a longer reaction time in the former condition.

Shift Task

Participants were presented with three stimuli that were each composed of one out of three features from three dimensions (pattern, color, shape). The combination of features changed from trial to trial. On each trial, participants chose one of the stimuli, which resulted in winning 1 or 0 points. On each trial, one feature was more likely to be rewarded than the other two (e.g. red), resulting in a point 75% of the time the participant chooses the relevant stimulus, compared to 25% of the time for the other two stimuli. This relevant feature stayed consistent for

15-25 trials, and then switched with no external cue to the participant. Thus the participant must infer that the most rewarding feature has changed based on feedback, and relearn the important feature.

The simplest DV was the overall accuracy on the task (chance being 33%). The task was also analyzed using logistic regression and and reinforcement learning (RL) model. The logistic regression modeled the probability of a correct response using the following equation:

$$(2)\ P(correct) = trial\ since\ switch * trial\ \#$$

The main effect of trials since switch was taken as a measure of learning speed, while the interaction was taken as a measure of "learning to learn".

The RL model from [47] was used to model trial-by-trial performance. This model learned an "attention" weight for different features which was updated based on feedback and informed future choices. These attention weights decayed over time. Three DVs were extracted from this model: β (inverse temperature) from the softmax decision function, η (learning rate) for the attention weight updates, and d (decay rate) for the attention weights.

Simon Task

Participants responded using one of two arrow buttons depending on the color of the box they saw on the screen. In the congruent condition, the side of the screen matched the response button, while in the incongruent condition it did not. Participants completed fifty trials for each condition.

Using trial-by-trial reaction time and accuracies, we calculated individual DDM parameters (drift rate, threshold and non-decision time) using HDDM fit across all participants. In addition, we fit the HDDM with "simon condition" (whether the stimulus was on the same side as the response arrow) as a categorical predictor of drift rate. The simon task is primarily analyzed in terms of this effect: response were faster when the stimulus was on the same side as the response key. Differences in drift rate between simon conditions (congruent - incongruent) was the measure of the "simon effect".

Simple Reaction Time

Participants were instructed to respond as quickly as possible when they saw an "X" on the screen. They completed three blocks of fifty trials. Average reaction time was the only DV.

Spatial Span

On each trial, participants saw a grid of squares then a sequence of squares flashed red. Participants were asked to indicate the sequence that flashed in the order of presentation for half of the trials and in the reverse order for the other half of the trials. They completed 14 trials per condition and received feedback after each trial. The sequence length started at 3 and increased by 1 if the participant entered the correct sequence. The number of digits decreased by 1 after two incorrect responses. The forward and reverse span were used as the two DVs for this task.

Stimulus Selective Stop Signal Task

On each trial, participants were shown one out of four stimulus, where each stimuli was associated with one of two responses, either the left or right hand. Participants were instructed to respond to the stimuli as quickly as possible without sacrificing accuracy. After initial practice to familiarize participants with the stimulus-response mapping, subjects were instructed that a blue star (stop signal) or an orange star ("ignore" signal) would appear on some trials. Participants were instructed to stop their response if they saw a blue star but not an orange star.

The stars appeared around the stimulus with a delay (stop-signal delay) ranging from 0ms - 850ms after stimulus onset. Stop-signal delay was adjusted during 'stop' trials using a one-up, one-down staircase procedure in 50ms increments. Participants completed 5 blocks of 60 trials each. 60% of the trials were "go" trials, 20% were "stop" trials (where the stop signal was shown for the critical hand), and 20% were "ignore" trials (where the stop signal was shown for the non-critical hand).

Stop signal reaction time was calculated based on the "go" and "stop" trials, a measure that putatively reflects inhibitory control. Using trial-by-trial reaction time and accuracies on the "go" trials, we also calculated individual DDM parameters (drift rate, threshold and non-decision time) using HDDM fit across all participants. In addition, we fit the HDDM with non-stop conditions ("go" vs "ignore") as a categorical predictor of drift rate. Reactive control was defined as the difference in drift rate between the non-stop conditions (ignore - go). Drift rate was expected to be smaller in the former condition, and greater in the latter condition, analogous to a longer reaction time in the former condition.

Stop Signal Task

On each trial, participants were shown one out of four stimulus, where each stimuli was associated with one of two responses, either the left or right hand. Participants were instructed to respond to the stimuli as quickly as possible without sacrificing accuracy. After initial practice to familiarize participants with the stimulus-response mapping, subjects were instructed that a red star (stop signal) would appear on some trials. Participants were instructed to stop their response if they saw a red star.

The star appeared around the stimulus with a delay (stop-signal delay) ranging from 0ms - 850ms after stimulus onset. Stop-signal delay was adjusted during 'stop' trials using a one-up, one-down staircase procedure in 50ms increments.

This task had two conditions which differed based on how frequent stop trials were (40% or 20% of trials). Participants completed 5 blocks of 60 trials each for each condition (the order of the two conditions was randomized across participants).

Stop signal reaction time was calculated separately for each condition. Proactive SSRT speeding was also calculated as the difference in SSRT between the two conditions (20% - 40%). Using trial-by-trial reaction time and accuracies on the "go" trials, we also calculated individual DDM parameters (drift rate, threshold and non-decision time) using HDDM fit across all participants. In addition, we fit the HDDM with condition (20% vs 40%) as a categorical predictor of drift rate and threshold. We allowed threshold to change as a function of condition because the stop frequency condition was a blocked change (rather than restricted to a particular trial), potentially causing strategic shifts in decision processing, reflected by a changed threshold. Proactive slowing was calculated as the difference in drift rate and threshold between the two

conditions (40% - 20%). Drift rate was expected to be smaller in the former condition, and greater in the latter condition, analogous to a longer reaction time in the former condition.

Stroop

Participants were instructed to respond using one of three keys depending on the ink color of the word that were presented. In the congruent condition, the word matched the ink color and in the incongruent condition they conflicted. There were 96 trials (8 repetitions of each of 6 incongruent pairs and 16 of each of 3 congruent pairs, resulting in 50% congruent trials).

Using trial-by-trial reaction time and accuracies, we calculated individual DDM parameters (drift rate, threshold and non-decision time) using HDDM fit across all participants. In addition, we fit the HDDM with condition (whether the stimulus color was congruent with the word) as a categorical predictor of drift rate. The stroop task is primarily analyzed in terms of this effect: responses were faster when the stimulus ink color was the same as the word. Differences in drift rate between congruent and incongruent trials (incongruent - congruent) was the measure of the stroop effect. Drift rate was expected to be smaller in the former condition, and greater in the latter condition, analogous to a longer reaction time in the former condition.

Cue/Task-switching Task

On each trial, participants saw a cue followed by a colored number (1-9) and responded to the colored number based on the task elicited by the cue. The 3 tasks were to judge the upcoming colored number based on: color (orange or blue), magnitude (greater or less than 5), or parity (odd or even). Each task was elicited by two cues (e.g. "orange-blue" or "color" would be

the cues for the task, color.). One response from each of the three tasks was associated with one button, while the other responses from each of the three tasks were associated with another button (e.g., press Z key if colored number was orange, greater than 5, or odd, depending on cue).

Cues for each task appeared above the stimulus on each trial. From trial to trial, the task and cue could stay the same, the task could stay the same and the cue could switch, or the task could switch (necessitating a cue switch). In addition, on task switch trials, the task could either switch to the last task ("task-switch-old", e.g. "color" -> "parity" -> "color") or to a new task ("task-switch-new", e.g. "color" -> "parity" -> "magnitude"). Thus there are four trial types which were randomly sampled across trials according to the following probabilities: task-switch-old (33%), task-switch-new (33%), task-stay-cue-switch (16.5%), and task-stay-cue-stay (16.5%). The cue-target-interval (CTI) was short (100ms) for half of the trials and long (900ms) for the other half. Participants completed 60 practice trials and 440 test trials.

Using trial-by-trial reaction time and accuracies, we calculated individual DDM parameters (drift rate, threshold and non-decision time) using HDDM fit across all participants. In addition, we fit the HDDM with cue condition (switch or stay), task condition (switch or stay) and CTI (100ms or 900ms) as categorical predictors of drift rate. Note that any time there was a task-switch, there was also a cue-switch. Differences in drift rate based on the cue condition (switch - stay) and task condition (switch - stay) were used as additional DVs. In both cases, drift rate was expected to be smaller in the former condition, and greater in the latter condition, analogous to a longer reaction time in the former condition.

Tower of London

On each trial, participants were presented with two boards: their board and a target board. Each board contained three balls dispersed across three pegs. Participants were instructed to make their board look like the target board by rearranging the colored balls while making as few moves as possible. Participants could move only one ball at a time and were instructed to plan their moves before execution. Each trial was capped at 20 seconds. Participants completed 12 trials of increasing difficulty (the optimal number of moves varied from 2 to 5).

Four DVs were calculated based on this task: average planning time (the time before the first move was initiated), average movement time (the average trial time excluding planning time), number of optimal solutions, and number of extra moves made beyond the optimal number.

Two-step Task

Participants made two sequential decisions between abstract shapes overlaid on different colored backgrounds. The first decision (Stage 1) between the two abstract shapes lead to one of two second "stages" (Stage 2 or Stage 3) where the participants made a second decision between two shapes. The decision in the second phase resulted in either winning a coin or not. Participants' goal was to win as many coins as possible. They were told that each shape in the first stage was more likely to lead to one second stage than the other and that these probabilities remain the same across the task. They were also told that the probabilities of winning a coin from choosing either shape in the second stage changed across the task. Participants completed 50 practice trials and 200 test trials. Total points on this task contributed to the final bonus payment.

Importantly, the task was structured such that each first-step decision lead to one second-stage (set of 2 shapes) frequently (70% of the time), and the other second-stage infrequently (30%). For instance, one shape in Stage 1 lead to Stage 2 frequently and Stage 3 infrequently. This task structure was stable throughout the experiment. On the other hand, reward probabilities associated with the Stage 2 and 3 shapes adjusted gradually and continuously over the experiment, to incentivize continued learning. Thus to perform optimally at the task, a participant must learn the transition probabilities at the first stage, and use them combined with trial-by-trial updates of reward probabilities to make optimal decisions.

Three DVs were calculated based on the following logistic regression:

Equation 2

$$P(stay)_t = feedback_{t-1} * transition_{t-1}$$

That is, the probability of making the same choice at *t* was modeled as a function of the interaction between feedback at *t-1* and the transition (frequent or infrequent) at *t-1*. A "model-free" index was calculated as the main effect of feedback, a "model-based" index was calculated as the interaction between feedback and transition, and a "perseverance" index was the intercept of the model. We used mixed-effects logistic regression using the lme4 R package [72] with the full interactive model fit as a random effect across participants. Individual DVs were defined based on these random effects.

Writing Task

Participants were asked to respond to the question “What happened in the last month?” for five minutes. They were asked to write for the whole time period and to stay on task. The task automatically ended after five minutes.

This text was minimally analyzed. We used a sentiment API created at text-processing.com to evaluate the text. This returned a probability of a "positive" and "neutral" classification. Though the exact relationship between classification probability and intensity was unclear, we used these probabilities as our only two DVs extracted from this task.

Description of Outcome Measures

The outcome measures were composed of surveys designed by others to assess particular real-world behaviors (see below) and a set of items specific to this work. The items specific to this work related to age, sex, weight, height, race, ethnicity, education level, relationship status, divorce count, years in a relationship, number of relationships, number of children, household income, debt, retirement account, whether they own or rent a house, traffic tickets, traffic accidents, past or current problems with gambling, caffeine intake, and legal troubles. The specific items and coding can be found at the expfactory-survey page, and the survey subscale scoring (the particular items used for each subscale) can be found within the expfactory-analysis repo. Data on individual surveys can be found in the Self Regulation Ontology repo.

Alcohol, Smoking and Drug Questionnaire

Participants were presented with a questionnaire assessing smoking, drinking, marijuana and other drug habits. The questions for alcohol are taken from the Alcohol Use Disorders

Identification Test (AUDIT [73]). The questions for smoking are adapted from questions used for the National Adult Tobacco Survey. The questions for marijuana are taken from Cannabis Use Disorder Identification Test - Revised (CUDIT-R [74]). The questions for other drugs are taken from the Drug Abuse Screening Test (DAST-10).

Kessler Psychological Distress Scale (K6+)

The Kessler Psychological Distress Scale (K6+)[75] is a 6-item self-report measure of psychological distress intended to be used as a quick tool to assess risk for serious mental illness in the general population. On the first critical item, participants indicate how often they have had six different feelings or experiences during the past 30 days using a 5-point Likert scale. The feelings and experiences for this first item are the following: “nervous,” “hopeless,”, “restless or fidgety,” “so depressed that nothing could cheer you up,” “that everything was an effort,” and “worthless.” The next item assesses the extent to which the feelings are typical for the person. The remaining items assess to what extent these experiences led to functional impairment.

Stanford Leisure-Time Activity Categorical Item (L-Cat)

The L-Cat [76] is a single item that is intended to measure people’s activity level. It provides six descriptions ranging from "I did not do much physical activity. I mostly did things like watching television, reading, playing cards, or playing computer games. Only occasionally, no more than once or twice a month, did I do anything more active such as going for a walk or playing tennis." to "Almost daily, that is five or more times a week, I did vigorous activities such

as running or riding hard on a bike for 30 minutes or more each time.” Subjects chose which description best fit their activity level.

Supplementary Discussion

Robustness Analysis for Hierarchical Clustering

Across the 5000 simulations, clustering solutions were modestly stable, with surveys showing greater stability (M±SD adjusted mutual information (AMI) across simulations = .78±.07), than tasks (AMI = .68±.05). The consensus clustering solution likewise was moderately related to the clustering solutions reported in the main results (survey AMI = .97, task AMI = .80)

We also calculated the percentage of simulations in which pairs of DVs were clustered together, and used the resulting co-occurrence matrix to provide another picture of cluster stability. Here we report summary metrics from this co-occurrence analysis, but it should be noted that individual DV pairs and whole clusters varied considerably in their co-occurrence strength. The online Jupyter Notebook presents the full co-occurrence matrix . Overall, co-occurrence between DVs within-clusters (survey/task M = .87/.70) was greater than co-occurrence across-clusters (M = .03/.04) and greater than co-occurrence with DVs from the two closest clusters (M = .08/.13).

Prediction using Individual DVs

Cross-validated results with individual DVs (i.e. without dimensionality reduction) are qualitatively the same as the EFA analysis using ridge regression. Lasso also showed qualitatively similar results, though quantitatively differed on specific targets. In particular, the target factor "Binge Drinking" was better predicted by Lasso with the survey DVs (R^2 = .25) than using the survey factor scores (R^2 = .13). Due to the variable selection imposed by lasso, only four DVs contributed to this prediction (TFEQ-R18: Cognitive Restraint, SSS: Disinhibition, ZTPI: Past Positive, DOSPERT: Healthy Safety Risk-Taking). This demonstrates a difficulty inherent in building predictive models using individual DVs - it is difficult to know how to generalize predictive success or how to connect prediction to theoretical constructs. While this again highlights the utility in making use of ontological factors, it also demonstrates that if prediction is the only goal, there are times when dimensionality reduction is deleterious. The full prediction results for both linear models using the DVs as predictors is shown in Supplementary Table 2. Overall, the qualitative agreement between prediction results with or without using EFA indicates that EFA did not generally remove information pertinent for outcome prediction.

Supplementary References

1. Patton, J. H., Stanford, M. S. & Barratt, E. S. Factor structure of the Barratt impulsiveness scale. *J. Clin. Psychol.* **51**, 768–774 (1995).
2. Carver, C. S. & White, T. L. Behavioral inhibition, behavioral activation, and affective responses to impending reward and punishment: The BIS/BAS Scales. *J. Pers. Soc. Psychol.* **67**, 319 (1994).

3. Roth, R. M., Isquith, P. K. & Gioia, G. A. *BRIEF-A: Behavior Rating Inventory of Executive Function--adult Version : Professional Manual*. (Psychological Assessment Resources, 2005).

4. Dickman, S. J. Functional and dysfunctional impulsivity: personality and cognitive correlates. *J. Pers. Soc. Psychol.* **58**, 95–102 (1990).

5. Blais, A.-R. & Weber, E. U. A Domain-Specific Risk-Taking (DOSPERT) Scale for Adult Populations. (2006).

6. de Lauzon, B. *et al.* The Three-Factor Eating Questionnaire-R18 is able to distinguish among different eating patterns in a general population. *J. Nutr.* **134**, 2372–2380 (2004).

7. Gross, J. J. & John, O. P. Individual differences in two emotion regulation processes: implications for affect, relationships, and well-being. *J. Pers. Soc. Psychol.* **85**, 348–362 (2003).

8. Baer, R. A., Smith, G. T., Hopkins, J., Krietemeyer, J. & Toney, L. Using Self-Report Assessment Methods to Explore Facets of Mindfulness. *Assessment* **13**, 27–45 (2006).

9. Carstensen, L. L. & Lang, F. R. Future time perspective scale. *Unpublished manuscript, Stanford University* (1996).

10. Duckworth, A. L. & Quinn, P. D. Development and Validation of the Short Grit Scale (Grit – S). *Journal of Personality Assessment* **91**, 166–174 (2009).

11. Eysenck, S. B. G., Pearson, P. R., Easting, G. & Allsopp, J. F. Age norms for impulsiveness, venturesomeness and empathy in adults. *Pers. Individ. Dif.* **6**, 613–619 (1985).

12. Brown, K. W. & Ryan, R. M. The benefits of being present: mindfulness and its role in psychological well-being. *J. Pers. Soc. Psychol.* **84**, 822 (2003).

13. Patrick, C. J., Curtin, J. J. & Tellegen, A. Development and validation of a brief form of the

Multidimensional Personality Questionnaire. *Psychol. Assess.* **14**, 150–163 (2002).

14. Baltes, P. B., Baltes, M. M., Freund, A. M. & Lang, F. R. The measure of selection, optimization, and compensation (SOC) by self-report. *Max Planck Institute for Human Development, Berlin* (1999).
15. Zuckerman, M. The sensation seeking scale V (SSS-V): Still reliable and valid. *Pers. Individ. Dif.* **43**, 1303–1305 (2007).
16. Carey, K. B., Neal, D. J. & Collins, S. E. A psychometric analysis of the self-regulation questionnaire. *Addict. Behav.* **29**, 253–260 (2004).
17. Gosling, S. D., Rentfrow, P. J. & Swann, W. B., Jr. A very brief measure of the Big-Five personality domains. *J. Res. Pers.* **37**, 504–528 (2003).
18. Job, V., Dweck, C. S. & Walton, G. M. Ego Depletion—Is It All in Your Head? *Psychol. Sci.* **21**, 1686–1693 (2010).
19. Zimbardo, P. G. & Boyd, J. N. Putting time in perspective: A valid, reliable individual-differences metric. in *Time Perspective Theory; Review, Research and Application* 17–55 (Springer, 2015).
20. Lynam, D. R., Smith, G. T., Whiteside, S. P. & Cyders, M. A. The UPPS-P: Assessing five personality pathways to impulsive behavior. *West Lafayette, IN: Purdue University* (2006).
21. Harvey, P.-O. *et al.* Cognitive control and brain resources in major depression: an fMRI study using the n-back task. *Neuroimage* **26**, 860–869 (2005).
22. Jaeggi, S. M., Buschkuehl, M., Jonides, J. & Perrig, W. J. Improving fluid intelligence with training on working memory. *Proceedings of the National Academy of Sciences* **105**, 6829–6833 (2008).
23. Pleskac, T. J. Decision making and learning while taking sequential risks. *J. Exp. Psychol. Learn. Mem. Cogn.* **34**, 167–185 (2008).

24. Jentsch, J. D., Woods, J. A., Groman, S. M. & Seu, E. Behavioral characteristics and neural mechanisms mediating performance in a rodent version of the Balloon Analog Risk Task. *Neuropsychopharmacology* **35**, 1797–1806 (2010).

25. Fan, J., McCandliss, B. D., Fossella, J., Flombaum, J. I. & Posner, M. I. The activation of attentional networks. *Neuroimage* **26**, 471–479 (2005).

26. Koffarnus, M. N. & Bickel, W. K. A 5-trial adjusting delay discounting task: accurate discount rates in less than one minute. *Exp. Clin. Psychopharmacol.* **22**, 222 (2014).

27. Toplak, M. E., West, R. F. & Stanovich, K. E. Assessing miserly information processing: An expansion of the Cognitive Reflection Test. *Think. Reason.* **20**, 147–168 (2014).

28. Primi, C., Morsanyi, K., Chiesi, F., Donati, M. A. & Hamilton, J. The Development and Testing of a New Version of the Cognitive Reflection Test Applying Item Response Theory (IRT). *J. Behav. Decis. Mak.* **29**, 453–469 (2016).

29. Figner, B., Mackinlay, R. J., Wilkening, F. & Weber, E. U. Affective and deliberative processes in risky choice: age differences in risk taking in the Columbia Card Task. *J. Exp. Psychol. Learn. Mem. Cogn.* **35**, 709–730 (2009).

30. Hare, T. A., Camerer, C. F. & Rangel, A. Self-control in decision-making involves modulation of the vmPFC valuation system. *Science* **324**, 646–648 (2009).

31. Woods, D. L. *et al.* Improving digit span assessment of short-term verbal memory. *J. Clin. Exp. Neuropsychol.* **33**, 101–111 (2011).

32. Nee, D. E., Jonides, J. & Berman, M. G. Neural Mechanisms of Proactive Interference-Resolution. *NeuroImage* **38**, 740–751 (2007).

33. Figner, B. *et al.* Lateral prefrontal cortex and self-control in intertemporal choice. *Nat. Neurosci.* **13**, 538–539 (2010).

34. Otto, A. R., Skatova, A., Madlon-Kay, S. & Daw, N. D. Cognitive Control Predicts Use of

Model-based Reinforcement Learning. *J. Cogn. Neurosci.* **27**, 319–333 (2013).

35. Badre, D., Kayser, A. S. & D'Esposito, M. Frontal Cortex and the Discovery of Abstract Action Rules. *Neuron* **66**, 315–326 (2010).
36. Holt, C. A., Laury, S. K. & Others. Risk aversion and incentive effects. *Am. Econ. Rev.* **92**, 1644–1655 (2002).
37. Clark, L., Robbins, T. W., Ersche, K. D. & Sahakian, B. J. Reflection Impulsivity in Current and Former Substance Users. *Biol. Psychiatry* **60**, 515–522 (2006).
38. Miyake, A. *et al.* The unity and diversity of executive functions and their contributions to complex 'Frontal Lobe' tasks: a latent variable analysis. *Cogn. Psychol.* **41**, 49–100 (2000).
39. Yntema, D. B. Keeping track of several things at once. *Human Factors: The Journal of the Human Factors and Ergonomics Society* **5**, 7–17 (1963).
40. Kirby, K. N. & MarakoviĆ, N. N. Delay-discounting probabilistic rewards: Rates decrease as amounts increase. *Psychon. Bull. Rev.* **3**, 100–104 (1996).
41. Aron, A. R., Behrens, T. E., Smith, S., Frank, M. J. & Poldrack, R. A. Triangulating a Cognitive Control Network Using Diffusion-Weighted Magnetic Resonance Imaging (MRI) and Functional MRI. *Journal of Neuroscience* **27**, 3743–3752 (2007).
42. Frank, M. J., Seeberger, L. C. & O'Reilly, R. C. By Carrot or by Stick: Cognitive Reinforcement Learning in Parkinsonism. *Science* **306**, 1940–1943 (2004).
43. Pashler, H. Dual-task interference in simple tasks: data and theory. *Psychol. Bull.* **116**, 220 (1994).
44. Raven, J. & Others. Raven progressive matrices. in *Handbook of nonverbal assessment* 223–237 (Springer, 2003).
45. Stahl, C. *et al.* Behavioral components of impulsivity. *J. Exp. Psychol. Gen.* **143**, 850–886 (2014).

46. Wilson, R. C. & Niv, Y. Inferring Relevance in a Changing World. *Front. Hum. Neurosci.* **5**, 1–14 (2012).

47. Radulescu, A., Daniel, R. & Niv, Y. The effects of aging on the interaction between reinforcement learning and attention. *Psychol. Aging* **31**, 747–757 (2016).

48. Lu, C.-H. & Proctor, R. W. The influence of irrelevant location information on performance: A review of the Simon and spatial Stroop effects. *Psychon. Bull. Rev.* **2**, 174–207 (1995).

49. Bissett, P. G. & Logan, G. D. Selective stopping? Maybe not. *J. Exp. Psychol. Gen.* **143**, 455 (2014).

50. Bissett, P. G. & Logan, G. D. Balancing cognitive demands: control adjustments in the stop-signal paradigm. *J. Exp. Psychol. Learn. Mem. Cogn.* **37**, 392–404 (2011).

51. Schneider, D. W. & Logan, G. D. Task-switching performance with 1:1 and 2:1 cue-task mappings: not so different after all. *J. Exp. Psychol. Learn. Mem. Cogn.* **37**, 405–415 (2011).

52. Shallice, T. Specific impairments of planning. *Philosophical Transactions of the Royal Society of London, Biology* **298**, 199–209 (1982).

53. Daw, N. D., Gershman, S. J., Seymour, B., Dayan, P. & Dolan, R. J. Model-based influences on humans' choices and striatal prediction errors. *Neuron* **69**, 1204–1215 (2011).

54. Eisenberg, I. W. *et al.* Applying novel technologies and methods to inform the ontology of self-regulation. *Behav. Res. Ther.* **101**, 46–57 (2018).

55. Sochat, V. V. *et al.* The Experiment Factory: Standardizing Behavioral Experiments. *Front. Psychol.* **7**, 610 (2016).

56. Henmon, V. A. C. The relation of the time of a judgment to its accuracy. *Psychol. Rev.* **18**, 186–201 (1911).

57. Wiecki, T. V., Sofer, I. & Frank, M. J. HDDM: Hierarchical Bayesian estimation of the

Drift-Diffusion Model in Python. *Front. Neuroinform.* **7**, 14 (2013).

58. Ratcliff, R. & Childers, R. Individual differences and fitting methods for the two-choice diffusion model of decision making. *Decisions* **2**, Advance online publication (2015).
59. Katahira, K. How hierarchical models improve point estimates of model parameters at the individual level. *J. Math. Psychol.* **73**, 37–58 (2016).
60. Stekhoven, D. J. & Buhlmann, P. MissForest--non-parametric missing value imputation for mixed-type data. *Bioinformatics* **28**, 112–118 (2012).
61. Enkavi, A. Z. *et al.* Large-scale analysis of test--retest reliabilities of self-regulation measures. *Proceedings of the National Academy of Sciences* **116**, 5472–5477 (2019).
62. Duckworth, A. L. & Quinn, P. D. Development and Validation of the Short Grit Scale (Grit–S). *J. Pers. Assess.* **91**, 166–174 (2009).
63. Whiteside, S. P. & Lynam, D. R. The Five Factor Model and impulsivity: using a structural model of personality to understand impulsivity. *Pers. Individ. Dif.* **30**, 669–689 (2001/3).
64. Zuckerman, M. Dimensions of sensation seeking. *J. Consult. Clin. Psychol.* **36**, 45 (1971).
65. Enkavi, A. Z. *et al.* A large scale analysis of test-retest reliabilities of self-regulation measures. *Manuscript In Preparation* (2018).
66. Lejuez, C. W. *et al.* Evaluation of a behavioral measure of risk taking: the Balloon Analogue Risk Task (BART). *J. Exp. Psychol. Appl.* **8**, 75–84 (2002).
67. Chandler, J., Mueller, P. & Paolacci, G. Nonnaïveté among Amazon Mechanical Turk workers: consequences and solutions for behavioral researchers. *Behav. Res. Methods* **46**, 112–130 (2014).
68. Rosvold, H. E., Mirsky, A. F., Sarason, I., Bransome, E. D., Jr & Beck, L. H. A continuous performance test of brain damage. *J. Consult. Psychol.* **20**, 343 (1956).
69. Servan-Schreiber, D., Cohen, J. D. & Steingard, S. Schizophrenic deficits in the processing

of context. A test of a theoretical model. *Arch. Gen. Psychiatry* **53**, 1105–1112 (1996).

70. MacDonald, A. W., 3rd *et al.* A convergent-divergent approach to context processing, general intellectual functioning, and the genetic liability to schizophrenia. *Neuropsychology* **19**, 814–821 (2005).
71. Toubia, O., Johnson, E., Evgeniou, T. & Delquié, P. Dynamic Experiments for Estimating Preferences: An Adaptive Method of Eliciting Time and Risk Parameters. *Manage. Sci.* **59**, 613–640 (2013).
72. Bates, D., Mächler, M., Bolker, B. & Walker, S. Fitting Linear Mixed-Effects Models using lme4. *arXiv [stat.CO]* (2014).
73. Saunders, J. B., Aasland, O. G., Babor, T. F., de La Fuente, J. R. & Grant, M. Development of the Alcohol Use Disorders Identification Test (AUDIT): WHO Collaborative Project on Early Detection of Persons with Harmful Alcohol Consumption-II. *Addiction* **88**, 791–804 (1993).
74. Adamson, S. J. *et al.* An improved brief measure of cannabis misuse: the Cannabis Use Disorders Identification Test-Revised (CUDIT-R). *Drug Alcohol Depend.* **110**, 137–143 (2010).
75. Kessler, R. C. *et al.* Short screening scales to monitor population prevalences and trends in non-specific psychological distress. *Psychol. Med.* **32**, 959–976 (2002).
76. Kiernan, M. *et al.* The Stanford Leisure-Time Activity Categorical Item (L-Cat): a single categorical item sensitive to physical activity changes in overweight/obese women. *Int. J. Obes.* **37**, 1597–1602 (2013).
